# Valence–arousal interactions between images and music: differential effects on memorisation, discrimination, and fixations

**DOI:** 10.7717/peerj.20781

**Published:** 2026-04-21

**Authors:** David Du Bruyn, Lena Esther Ptasczynski, Jochen Steffens

**Affiliations:** 1 Audio Communication Group, Technische Universität Berlin, Berlin, Germany; 2 Berlin School of Mind and Brain, Humboldt Universität Berlin, Berlin, Germany; 3 Department of Psychiatry and Neurosciences, Charité-Universitätsmedizin, Corporate member of Freie Universität Berlin and Humboldt-Universität zu Berlin, Berlin, Germany; 4 Institute of Sound and Vibration Engineering, Hochschule Düsseldorf, Düsseldorf, Germany

**Keywords:** Valence, Arousal, Memory, Congruence, Affective priming, Visual attention, Recognition memory, Valence arousal interaction, d-prime

## Abstract

Prior research has reported differential roles of valence and arousal in guiding memory and attention. However, few studies have systematically examined how these affective dimensions interact across their full spectrum in audiovisual contexts, particularly when multiple images varying in emotional content are simultaneously present. To address this, we used a recognition memory paradigm in which participants viewed arrays of images representing all combinations of positive/negative valence and low/high arousal. Slightly before and during image presentation, music conveying an emotional tone, also varying in valence and arousal, was played. Memory accuracy was assessed in a later recognition phase. We further employed simple eye-tracking measures to explore how visual attention is influenced by image and music valence and arousal. Results revealed that high image arousal increases fixation duration, whilst high music arousal decreases fixation duration. Memorisation likelihood was not influenced by a four-way interaction of image and music valence and arousal, but mainly by an interaction of image valence and arousal, differently depending on music arousal. In high music arousal, all images except low arousal positive images, were memorised regardless of valence. In low arousal music, we observed that memorisation likelihood was mainly driven by high image arousal, but only paired with negative image valence was memorisation significantly higher compared to other image types. Discrimination accuracy was not observed to be influenced by image valence and arousal, but positive high arousal music significantly improved memory discrimination. By systematically manipulating both valence and arousal of images and music, we highlight how the interaction of these unimodal affective qualities can facilitate or hinder memory.

## Introduction

Human core affect as well as the affective qualities of events and objects can be described by two orthogonal dimensions, the pleasantness-unpleasantness valence dimension and the activation-deactivation arousal continuum. In respect to memory and attention, valence and arousal seem to be processed differently (*e.g.*, Kensinger, 2004; Kensinger & Corkin, 2004) and different effects of valence and arousal processing, especially for high arousing stimuli across the valence dimension, have been demonstrated, potentially rendering the effect of arousal valence-dependent (Mickley Steinmetz, Addis & Kensinger, 2010). This study investigates how the interaction of image and music valence and arousal influence memory for images primed by affective music, and explores potential links to visual attention. We controlled for the affective qualities of the stimuli by pretesting each for valence and arousal, allowing us to directly examine the unimodal and interactive effects of these dimensions.

The role of arousal in memory can overall be seen as strengthening, with generally facilitated long-term memory for high arousal stimuli (Storbeck & Clore, 2008; Bradley et al., 1992). However, findings become more complex when arousal is considered in interaction with valence. Greene, Bahri & Soto (2010) in a recognition memory test for neutral shapes, played either of four different music types as means of mood induction, varying in the combination of high/low arousal and positive/negative valence, between a study and a recognition phase. The authors found increased memory sensitivity in positive high arousal and negative low arousal conditions, suggesting a dependence of the effect of arousal on valence. In contrast, Bradley et al. (1992), in a free recall test for images, found no such difference for high arousal stimuli across the valence dimension. After a period of one year, high arousal images regardless of valence, showed high memory performance compared to low arousal images. Only in the immediate free-recall, did valence have a small and marginally significant influence, with positive arousing images remembered slightly better than negative arousing images. In both memory tasks and tasks primed by affective content, valence and arousal interact, with high arousal consistently showing beneficial effects. In a word evaluation task, Zhang, Kong & Jiang (2012) primed participants with affective images, either valence congruent or valence incongruent to the target word. They found that arousal modulated affective priming, with valence congruence effects stronger with high arousing primes. Responses in valence congruent conditions were faster, compared to incongruent conditions, when the prime picture was high arousing and they concluded that affective priming was strongest when at least one of the stimuli (target or prime) was highly arousing, as supported by both reaction time and accuracy measures. Similarly, in a lexical decision task, asymmetric effects of valence in priming were found by Yao et al. (2019). Positive primes consistently facilitated responses to congruent targets, whilst negative primes slowed reaction times for congruent targets. Arousal priming occurred when primes and target shared positive valence, suggesting that arousal effects depend on valence support for priming effects to occur reliably. Their experiment demonstrated a stable advantage of positive affect and underlines the relatedness of valence and arousal. While affective priming effects do not necessarily have to directly translate to memory, they offer insight into early affective processing dynamics that may later shape memory performance.

Additionally, high arousal is likely to be processed with higher priorization, regardless of attentional demands due to a secondary task (Kensinger & Corkin, 2004), and in an automatic fashion (Kensinger, 2004; Mickley Steinmetz, Addis & Kensinger, 2010), with attention being more easily captured and sustained by high arousal items (Kensinger, 2009). The arousal dimension can be seen as having an amplifying function for affective information across levels of valence and providing a sense of urgency (Storbeck & Clore, 2008). Accordingly, arousing stimuli have been shown to be remembered better than non-arousing stimuli (Kensinger, 2009). In the literature, extraneous arousal (arousal unrelated to the object under investigation) has been observed to enhance the perceived arousal of the target object. Chemically or electrically induced arousal can produce similar behavioural effects to stimulus-inherent or subject-inherent arousal, often leading to improved memory performance. Likewise, memory for objects has been shown to improve when arousal from unrelated sources is misattributed to the relevant object (see Bradley et al., 1992; Storbeck & Clore, 2008 for brief discussions).

Valence can be seen as a valuative relationship of a person with their surroundings and a ubiquitous invariant feature in affect and emotions (Barrett, 2006). The distinction between good and bad, approach or avoid, *etc*. is learned early in life and widely understood (Baumeister et al., 2001). In contrast to the relatively automatic processing of arousal, the valence dimension is understood to be more consciously processed, with elaboration or rehearsal processes, linking new to old information (Kensinger, 2004). These elaboration processes have been proposed to be especially involved in memory formation for low-arousing (positive) objects when attentional resources are not heavily demanded elsewhere. This effect was found to be diminished in situations where cognitive load is high, indicating that the availability of attentional resources plays a crucial role in facilitating these processes (Kensinger & Corkin, 2004). However, in the literature, differential effects for both ends of the valence continuum have been observed, with memory and attention benefits for positive stimuli (compare Pollyanna principle), as well as beneficial processing for negative items. Baumeister et al. (2001) for example discuss, that in general, “bad is stronger than good”, such as negative valenced events or objects will have larger impact than positive ones, hence strengthening the power of the negative. This negativity bias can result in a relative salience, due to aversiveness and avoidance or a need for change. However, in contrast positive stimuli and events can lead to approach tendencies and hence strengthen memory and attention for these. That is, positive affect has been observed to be related to improved cognition and problem solving (Greene, Bahri & Soto, 2010), with broader attentional focus and more heuristic processing (Gasper & Clore, 2002).

Despite being two seemingly orthogonal dimensions, valence and arousal are not completely unaffected by one another. As mentioned before, the effects of arousal have been partially found to be dependent on valence. Also, high arousal information can have a connotative tendency towards negative valence, whilst low arousing information can be positively connotated (Storbeck & Clore, 2008). This can be observed especially when portraying highly negative valenced information. From anecdotal evidence, thinking of highly negative information or events can release a sense of heightened arousal. This reflects in the spread of affective ratings in stimulus databases for the low arousal, negative valence quadrant in the two-dimensional valence-arousal space, such as in the EmoMadrid (Carretié et al., 2019), the OASIS (Kurdi, Lozano & Banaji, 2017) or the IAPS (Lang, Bradley & Cuthbert, 2005) image database, as well as for negative sounds in the IADS database (Yang et al., 2018), where a lack of very low arousal, highly negative ratings can be observed. This observation is corroborated by findings by Carretié et al. (2019) who found a significant correlation between valence and arousal, with arousal increasing as valence decreases.

In evaluative priming, the valuation of one stimulus influences the valuation of a subsequent stimulus (Barrett, 2006). In a typical affective priming paradigm, an affective prime (*e.g.*, a positive image) is presented with the subsequent presentation of a target (*e.g.*, a word). Participants are typically asked to rate the valence of a target or categorize the target as quick as possible as positive or negative. Congruence effects, such as a faster response for congruent targets, are often observed, with the valence congruence effect being well documented (Armitage & Eerola, 2022). This phenomenon has been studied across modalities, including sound to image processing (*e.g.*, Vallet, Brunel & Versace, 2010) and sound to word processing (*e.g.*, Steinbeis & Koelsch, 2011), with a few theories aiming to explain its mechanisms (see Scherer & Larsen, 2011 for a review). While both valence and arousal dimensions have been implicated in priming effects, the literature presents inconsistent findings regarding their relative roles. Whilst valence congruence effects are well-established, arousal congruence effects remain less well studied. Some studies suggest that valence plays a dominant role in priming (*e.g.*, Steinbeis & Koelsch, 2011), while others find arousal being the mainly transferred dimension (Marin, Gingras & Bhattacharya, 2012), again others argue for a differently weighted role of both valence and arousal in priming (*e.g.*, Scherer & Larsen, 2011) (see Armitage & Eerola, 2022 for a brief overview). As mentioned before, from a perspective of excitation transfer (Zillmann, 1971), arousal from unrelated sources, such as from arousing objects or circumstances, can be (mis-) attributed to a relevant object (Storbeck & Clore, 2008). This is not necessarily a congruence effect, as this extraneous arousal could be attributed to, and increasing the arousal of initially only mildly arousing relevant objects (Storbeck & Clore, 2008). A perspective on the role of arousal in priming, suggests that high arousal primes lead to vigilance processes, looking for further high arousing stimuli, irrespective of the valence of the information presented (Armitage & Eerola, 2022), thus rendering facilitation effects for negative and positively valenced conditions. The alarming nature of high arousal, signaling potential threat and a need for action, aligns with the narrowing of the attentional spotlight and shifts in cognitive concepts (Gasper & Clore, 2002) in narrowing on salient within object details, supporting this perspective. However, Armitage & Eerola (2022) report multiple studies from the literature, showing a lack of coherence for arousal congruence effects across different target modalities. The automatic fashion in the facilitation of memory for arousing stimuli and memory benefits for non-arousing materials, is reflected by the two distinct neural mechanisms described by Kensinger & Corkin (2004). The authors explain that memory enhancement for arousing material is associated with an amygdalar–hippocampal network, whilst negative but non-arousing material would be processed by a prefrontal-cortex–hippocampal network, which in contrast allows for more controlled memory processes. The proposal of a controlled process for valenced non-arousing material was corroborated by behavioural results, showing diminished memory benefits when a concurrent task demanded attentional resources. Two distinct routes of processing could hence be the root for the observed differential processing of valence and arousal in behavioural findings.

Although some studies carefully control valence and arousal, more data is needed for each specific combination of valence and arousal, such as low arousal with positive valence or high arousal with positive valence, to identify differential effects of these unimodal qualities and their interaction.

In the present study, we aimed to further clarify the interplay between valence and arousal in recognition memory by carefully controlling valence and arousal of the stimuli used by means of pre-testing. As music is strong in its influence on emotional states (Tesoriero & Rickard, 2012), we used music stimuli as auditory primes. We here employed a priming/recognition memory task, in which affective music excerpts primed image presentation, for which subsequent recognition memory was probed. We controlled the valence and arousal levels of both the primes and targets, encompassing four distinct combinations. By ensuring control over the affective qualities of our stimuli, we aimed to provide valuable insights into the seemingly interacting effects of valence and arousal on memory and attention and further add to the disambiguation of the partially incoherent findings in the literature.

As a basis for further investigation, we included simple eye-tracking measures to examine how visual attention is influenced by affective stimulus properties and their interactions across modalities.

Given the differential nature of the findings on the valence-arousal interplay in the literature on memory formation, we expected to find similarly differential effects of both dimensions across the different combinations of valence and arousal.

In line with theories of arousal being a rather automatically processed dimension, with an alerting function, we expected that high arousing images are significantly better remembered and discriminated compared to low arousal images, regardless of the prime music valence and arousal and irrespective of the valence and arousal rating of the accompanying music (H1). We used memorization likelihood (hit: whether a target image was correctly remembered) and memory discrimination accuracy (d’, d Prime) as dependent variables, respectively. In addition, we expected that high arousal images, are not only remembered/discriminated better, but that high arousal facilitates attention to these images. We therefore assumed that the arousal of an image compared to the valence of an image, significantly increases the average and total fixation duration of images (H2).

Concerning valence, we expected images matching the valence of the music (congruent) to show stronger memory facilitation compared to images matching music arousal only. We expected this in low arousal prime conditions, due to potentially more resources for elaborative processing in low arousal music context. This will likely occur with larger facilitation for negative-valenced images compared to positive images (H3). This effect should manifest as higher memorisation likelihood and d′ scores for images matching the music valence, particularly in low arousal music conditions, where attentional demands are lower. Even if there is an underlying effect of high image arousal, we still expected valence-congruent images, regardless of their arousal level, to result in higher d′ scores or a higher likelihood of being remembered compared to valence-incongruent images.

## Method and Materials

### Participants

Forty-three participants (25 male, 18 female) with a mean age of 28.7 years (SD = 11.9) took part in the experiment. They were recruited *via* mailing lists and were compensated with 10 €. Highest education as reported by the participants was distributed as follows: Middle School 13.95%, High School 72.1%, Bachelor’s or Master’s Degree 13.95%.

All participants provided informed written consent by confirming their agreement on-screen prior to participation. This study was approved by the ethics committee of the medical faculty of the University Duisburg–Essen (21-9986-BO).

### Image stimuli

We used 74 colour images in total, taken from the Open Affective Standardised Image Set (OASIS) (Kurdi, Lozano & Banaji, 2017) and from the EmoMadrid database (Carretié et al., 2019), as these images have been compiled to serve research in the affective sciences and are pre-rated along valence and arousal (see Table S1 for the list of images used). To roughly equate visual saliency in terms of brightness of the images, all images were processed to share the same mean RGB converted brightness from grayscale of 122.78, which corresponds approximately to a luminance of 60.45 cd/m^2^ on our screen (125.7 cd/m^2^ for a white screen).

As in our experiment the presentation times and brightness of the images were different to the values in the original norming studies, we conducted a pre-test with originally 96 brightness adjusted images, from which the final 74 images were chosen for the main experiment. In an online test, we presented images from four different semantic categories ‘humans’, ‘animals’, ‘scenes’, and ‘objects’. One hundred participants rated 32 randomly presented images presented for one second, along valence and arousal on a five-point Likert scale. Along the lines of the categorical perception model, which suggests that perception is more distinctly between categories compared to within categories (Estes & Adelman, 2008; Juslin & Laukka, 2003), we decided to binarize the valence and arousal ratings of our stimuli. Based on the ratings from the pre-test, we binarized the image ratings to either negative/positive valence (0/1) and low or high arousal (0/1). Next, to the ease of use, our binarized approach avoids possible noise from fine grained continuous data, while allowing us to capture the relevant distinctions.

Participants from the online pre-test did not take part in the laboratory test. The pre-test ratings showed no significant differences in arousal between high arousal positive and high arousal negative valenced images as well as between low arousal positive and low arousal negative valenced images. Furthermore, no significant differences in valence ratings were found between low arousal negative and high arousal negative images. However, we observed that participants rated low arousal positive images significantly less positive compared to high arousal positive valence images, as indicated by a linear mixed model with *t*-tests and 95% confidence intervals (β = 0.39, 95% CI [0.27–0.52], t(3164) = 8.191, *p* < 0.001).

Each image from the presentation phase was assigned a partner image, taken from our image pool, which was used in the recognition phase as a distractor. In the recognition phase of the trial, only half of the initially presented images (targets) were shown and six new images (distractors) were added. Each partner image was semantically related and equally rated in terms of valence and arousal, such as an image of dog and its partner image of a cat. In the recognition phase, it was randomly chosen whether the original image was presented as a target or the partner image as a distractor, so that each participant had an individually randomized set of targets and distractors.

The images were presented to the participants as a collage with two rows of six images, with three images from each valence-arousal combination and semantic category. Images had a size of 300 × 240 pixels and were presented in colour on a gray background which had the same mean luminance as the images. The luminance was kept constant across the whole experiment (60.45 cd/m^2^).

### Music stimuli

As music stimuli, we selected 12 music pieces from the study by Eerola & Vuoskoski (2011) and other sources (see Table S2 for the list of music used). The music excerpts used were trimmed to duration of 15 s. As with the images, we included the trimmed music pieces in the online pre-test to obtain valence and arousal ratings from 100 participants. Based on these ratings, we binarized the valence and arousal ratings of the music pieces to positive *vs.* negative valence (0/1) and low *vs.* high arousal (0/1).

### Procedure

The experiment was conducted in a dimly lit and acoustically treated room. Participants were seated at a table in front of a screen (Lenovo ThinkVision, 24”) with the Gazepoint GP-3 eye tracker (Gazepoint, Vancouver, BC, Canada; https://www.gazept.com/ product/gazepoint-gp3-eye-tracker/?v=5f02f0889301) positioned directly below the presentation screen. At the beginning of the main experiment, participants received an introduction to the experiment task without revealing the exact purpose of the experiment. They completed three test trials to make sure the instructions were understood correctly, followed by the calibration of the eye tracker. After that, they were asked about their current mood (measured by valence and arousal on a five point Likert scale) and answered basic demographic questions. Finally, they completed nine questions taken from the GoldMSI “Short Scale (3 Items)” (Müllensiefen et al., 2014) from the categories “Musical Training”, “Perceptual Abilities” and “Emotions” (not further mentioned in the remainder of the paper).

Each of the 36 trials of the main part of the experiment started with a gray background. After two seconds, a black fixation cross appeared in the middle of the screen for ten seconds. The fixation cross was presented so that the gaze of participants would always start at the same position. The time before stimulus presentation was used to obtain a seven second baseline measurement for the pupillometry. Five seconds after onset of the fixation cross, the music playback started. After the fixation cross offset an image collage was presented for eight seconds. We chose a priming paradigm to achieve a possibly enhanced sensitivity to the image stimuli, whilst providing a no audio condition as a control condition. We can therefore better distinguish between the effects of the individual music types as well as the overall effect of music from the effect of no music, with the latter allowing us to assess the effect of the visual stimuli alone.

**Figure 1:**
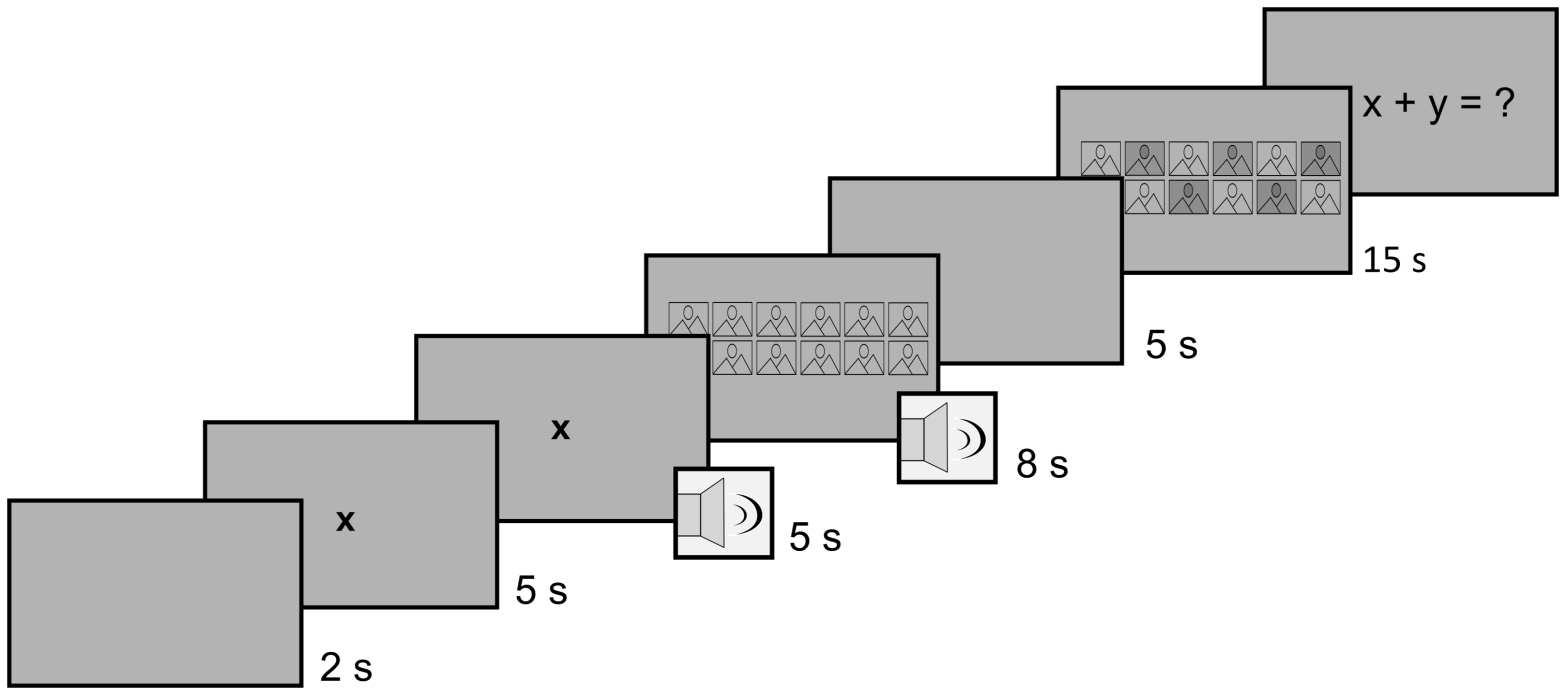
Timeline of a trial. The diagram shows the duration of each part of the trial in seconds.

A five second gray screen appeared after the music and image offset to reduce a possible recency bias. A 15 s recognition phase followed, in which participants were presented with a new image collage, consisting of six images from the presentation phase (targets) and six new images (distractors). Figure 1 shows a timeline of a single trial, illustrating the sequence and timing of stimulus presentation and response. Participants were asked to click all the images they recognized from the presentation phase and ignore all new images. After completion of the recognition phase, participants were presented with a simple arithmetic problem to solve. This was done to reduce carry-over effects between the trials. After completing all 36 trials, participants indicated their mood again along valence and arousal on a five-point Likert scale. Finally, they were debriefed and thanked for their participation.

### Pre-processing of eye tracking data

The raw eye tracking data were cleaned of outliers and parsed into fixations using the ‘saccades’ package (v. 0.1) (Von der Malsburg, 2015) for R (R Core Team, 2022). We analyzed the eye tracking data from the presentation phase of the experiment. As recommended by the author of the ‘saccades’ package, we set the fixation detection algorithm for use with a low frequency (60 Hz) eye tracker, with ‘smooth.coordinates = TRUE’ and ‘smooth.saccades = FALSE’ and a ‘lambda’ value of 6. Using the ‘eyetrackingR’ package (v. 0.2.1) (Forbes, Dink & Ferguson, 2023), we computed points of gaze within areas of interest (*i.e.,* the images of our collages).

### Pupillometry

To better understand whether the experimental task induced (physiological) arousal, we measured the pupil size during the priming and the presentation phase. The pupil data was processed using the ‘PupillometryR’ package (v. 0.0.5) according to the recommendations of its author (Forbes, 2020). The data was further cleaned by removing blinks and smoothed by regressing one pupil against the other. We generated a mean pupil value by computing the mean of both pupils, calculated the median pupil size per time bin, and subsequently filtered the data using an 11-degree median filter. We then baselined the pupil data to a seven-second baseline period, which occurred prior to the priming phase. Although this baseline period is longer than is conventionally used in pupillometry studies, it was chosen to maximize the signal-to-noise ratio and to mitigate three specific potential confounds: pupil dilation from cognitive load after the arithmetic task in the preceding trial, surprise effects due to the appearance of the fixation cross, and pupil dilation related to anticipation of the next trial. By averaging across the full seven seconds, we aimed to minimize these trial-specific effects and ensure a stable baseline for subsequent analysis. While this approach captures more general variability, we deemed this trade-off acceptable given the methodological constraints and the importance of reducing noise from the aforementioned confounds.

## Results

### Memorisation (hit)

To investigate how the interaction of image valence, image arousal, music valence and music arousal determines image memorisation likelihood, we fitted a generalized linear mixed effects model (GLMM) by maximum likelihood, implemented using the lme4 package (Bates et al., 2015) with binomial error distribution and logit link function. To ensure model convergence given the complex interaction structure, we used the bobyqa optimizer with 200,000 iterations. The four-way interaction of these valence and arousal ratings with all lower-order interactions and main effects of the individual predictors served as fixed effects as well as the mean pupil size per trial. A random intercept for subject was included to account for repeated observations within participants. The dependent variable ‘Hit’ at the image level indicated in binary form whether an image was remembered (1) or not (0). The image and music valence and arousal ratings were binarized as well to low *vs.* high arousal (0/1) and negative *vs.* positive valence (0/1). For analysis, these binary predictors were effect-coded (−0.5/+0.5) to allow interpretation of main effects as deviations from the grand mean.

Results from the Type II ANOVA (Wald *χ*^2^ tests) using the car package (v 3.1-3) (Fox & Weisberg, 2019) revealed significant main effects and interactions. Image valence (*χ*^2^(1) = 9.03, *p* = 0.002), as well as image arousal (*χ*^2^(1) = 37.90, *p* < 0.001) were significant, whilst music valence (*χ*^2^(1) = .37, *p* = 0.54) and music arousal (*χ*^2^(1) = 0.48, *p* = 0.48) showed no significant main effect on memorisation likelihood (‘hit’). The mean pupil size showed no significant influence (*χ*^2^(1) = 0.007, *p* = 0.93).

The four-way interaction of image valence, image arousal, music valence and music arousal was not significant (*χ*^2^(1) = 0.077, *p* = 0.78). The results however showed significant three-way interactions of image valence, image arousal and music arousal (*χ*^2^(1) = 10.49, *p* = 0.001) as well as image valence, music valence and music arousal (*χ*^2^(1) = 4.27, *p* = 0.03). The image valence and music valence two-way interaction was significant as well (*χ*^2^(1) = 4.97, *p* = 0.02). The model showed a low proportion of variance explained by random effects (adjusted ICC = 0.021) and overall low *R*^2^ (conditional *R*^2^ = 0.03; marginal *R*^2^ = 0.009).

We conducted a *post-hoc* analysis of estimated marginal means (emmeans) (v. 1.8.3) (Lenth, 2022) with Tukey adjustment for multiple comparisons, for both three-way interactions. The significance threshold was set at α = .05, and 95% confidence intervals were reported for all estimates and back-transformed from the log odds ratio scale.

### Image valence x image arousal x music arousal

Pairwise contrasts showed that, in low arousal music, positive arousing images were significantly more likely to be marked as remembered compared to negative valence low-arousing images (OR = 0.71, SE = 0.05, 95% CI [0.55–0.90], *z* = −4.28, *p* < 0.001) as well as compared to positive low arousing images (OR = 0.72, SE = 0.05, 95% CI [0.56–0.91], *z* = −4.16, *p* < 0.001).

In high-arousal music positive valence low-arousal images were significantly more likely to be marked as remembered, compared to negative valence low-arousal images (OR = 1.37, SE = 0.11, 95% CI [1.06–1.76], *z* = 3.77, *p* = 0.004).

**Figure 2:**
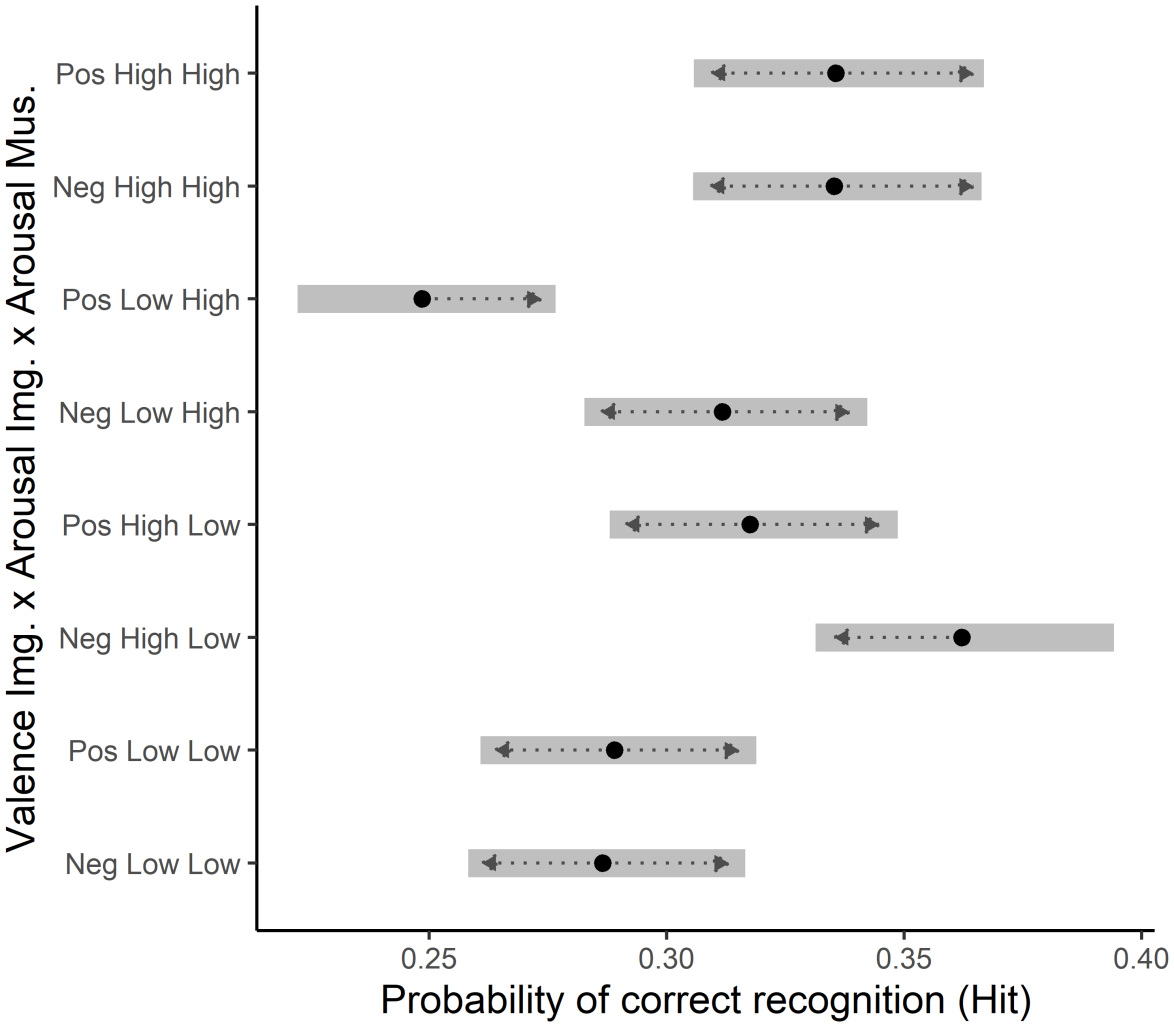
Estimated marginal means of image memorisation likelihood per condition. *Post-hoc* analysis of estimated marginal means of the image valence, image arousal, and music arousal interaction, with Tukey adjustment for multiple comparisons. Error bars represent 95% confidence intervals, back-transformed from the log odds ratio scale. Significance threshold was set at α = 0.05. Dotted lines represent comparison arrows; overlap indicates insignificant difference between two conditions.

Further, in high-arousal music, positive valence low-arousal images were more likely marked as remembered, compared to negative high-arousal images (OR = 0.65, SE = 0.05, 95% CI [0.51–0.84], *z* = −5.12, *p* < 0.001) as well as compared to positive high-arousing images (OR = 0.65, SE = 0.05, 95% CI [0.51–0.84], *z* = −5.13, *p* < 0.001). See Fig. 2 for a plot of the estimated marginal means of hit likelihood per condition.

### Image valence x music valence x music arousal interaction

*Post-hoc* pairwise comparisons for the three-way interaction of image valence, music valence, and music arousal showed several significant pairwise differences.

*Post-hoc* pairwise comparisons show, that across image arousal levels, negative images in negative valenced low arousing music trials were significantly more likely to be marked as hit, compared to both positive images in negative valenced low arousing music trials (OR = 1.31, SE = 0.10, 95% CI [1.02–1.67], *z* = 3.30, *p* = 0.02), and positive valenced images in negative valenced high arousing music (OR = 1.37, SE = 0.11, 95% CI [1.07–1.77], *z* = 3.84, *p* = 0.003).

**Figure 3:**
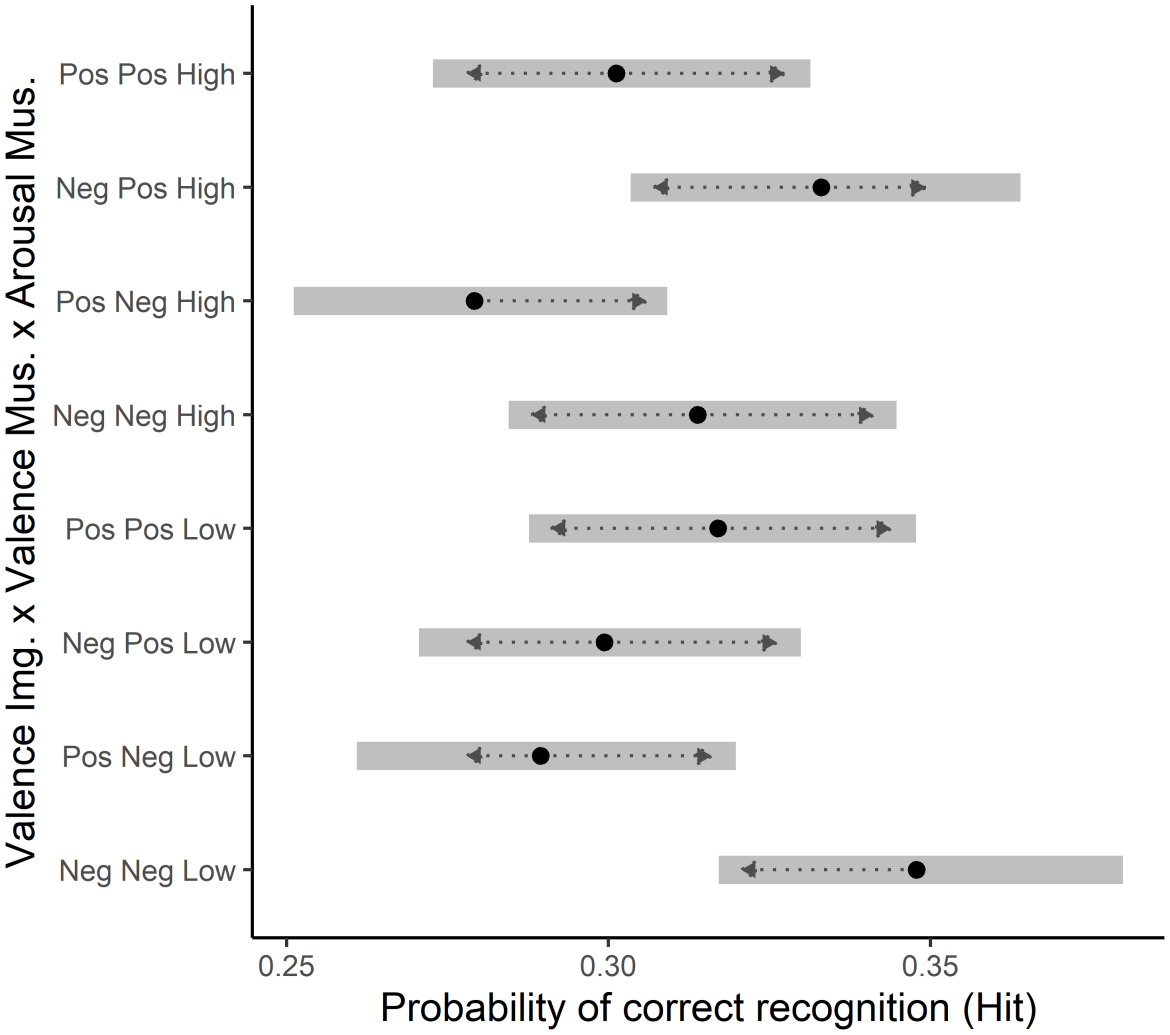
Estimated marginal means of image memorisation likelihood per condition. *Post-hoc* analysis of estimated marginal means of the image valence, music valence, and music arousal interaction, with Tukey adjustment for multiple comparisons. Error bars represent 95% confidence intervals, back-transformed from the log odds ratio scale. Significance threshold was set at α = 0.05. Dotted lines represent comparison arrows; overlap indicates insignificant difference between two conditions.

Additionally, positive valenced images in negative valenced high arousal music trials were significantly less likely to be marked as remembered, compared to negative valenced images in positive valenced high arousal music trials (OR = 0.77, SE = 0.063, 95% CI [0.60–0.99], *z* = −3.09, *p* = 0.041). See Fig. 3 for a plot of the estimated marginal means of hit likelihood per condition.

### Discriminability (d’)

To compliment the analysis of memorisation likelihood, we next analysed participants’ discrimination performance using the sensitivity measure of d’ (d prime). The trial level signal detection measure was computed from hit and false alarm rates. Higher values reflect more accurate discrimination between targets and distractors.

A linear mixed-effects model was used to estimate sensitivity (d′) as a function of image valence, image arousal, music valence, and music arousal and mean pupil size per trial. All binary predictors were effect-coded (−0.5/+0.5), and a four-way interaction was included as fixed effects. We included subjects as a random effect. Because residual diagnostics indicated deviation from normality, 2000 parametric bootstrap samples were used to obtain 95% confidence intervals for fixed effects.

Under this coding scheme, coefficients represent deviations from the grand mean of d′ across all experimental conditions. Bootstrapped confidence intervals indicated one reliable interaction: positive, high-arousal music was associated with higher d′ compared to negative low arousal music conditions (β = 0.108, 95% CI [0.030–0.186]). No other effects or interactions reached significance.

### Average fixation duration per image and trial

We examined how the four-way interaction of image valence, image arousal, music valence, and music arousal and all of its lower order interactions and main effects affect visual attention, specifically the average fixation duration per image and trial. We fitted a linear-mixed-effects model (LMM) with the log-transformed average fixation duration per trial for each image, as dependent variable. To address the positive skew in the fixation durations, we log-transformed the dependent variable. The four-way interaction of image valence, image arousal, music valence, and music arousal and a mean pupil size per trial variable served as fixed effects, and we included a random intercept for participants.

Due to violations of model assumptions (significant Kolmogorov–Smirnov test, Shapiro–Wilk test and patterned residuals) we here report the parametric bootstrapped confidence intervals (2,000 samples) to obtain robust estimates. The results revealed two significant main effects. High image arousal significantly increased the average fixation duration compared to low image arousal (β = 0.043, 95% CI [0.026–0.061]) and high music arousal was associated with significantly shorter average fixation durations (β =  − 0.03, 95% CI [−0.051 to −0.017]).

### Total fixation duration per image and trial

We computed a second LMM for the total fixation duration per image per trial, following the same specification as for the average fixation duration model. Due to violations of model assumptions, bootstrapped confidence intervals were computed. As observed in the average fixation duration results, high image arousal significantly increased the total fixation duration of an image per trial (β = 0.05, 95% CI [0.03–0.07]), whilst high music arousal significantly decreased total fixation duration per image per trial (β = −0.02, 95% CI [−0.04 to −0.005])

### Simulation based power analysis

To assess the sensitivity of our study to detect medium-sized effects and to estimate the sample size required for 80% statistical power, we conducted simulation-based power calculations for the most central effects of our study, using the simr package for R (Green & MacLeod, 2016). Power could not be reliably estimated for the d′ and fixation duration models due to computational issues, despite the flexibility of the simulation approach.

We specified the data-generating models for the simulations to match the structure of the fitted models derived from the observed data. For each target effect we set the hypothetical effect sizes to a medium effect (log-odds β = 0.5 for the binomial hit model). All simulations used α = 0.05 and models were fit with lme4. Due to computational restraints, we computed the power at our original sample size (*n* = 41) and at a hypothetical *n* = 100, to estimate the required n to achieve 80% power. Results are reported as the estimated power (proportion of simulated datasets with *p* < 0.05).

### Four-way interaction memorisation likelihood model

For the memorisation likelihood model, we evaluated the theoretical most important four-way interaction between image valence, image arousal, music valence and music arousal.

The estimated power (1,000 simulations) at our observed sample size (*n* = 41) was 34.70% (95% CI [31.8–37.7%]). Extending the model to a hypothetical *n* = 100 increased estimated power to 64.00% (95% CI [53.79%–73.36%]). Due to computational restraints, the power analysis for a sample size of 100 participants was computed with only with 100 simulations. These results indicate that this study is underpowered to detect a medium-sized four-way interaction at our current sample size, and null interactions should therefore be interpreted with caution. Achieving 80% power or more would require substantially more participants.

### Three-way interactions memorisation likelihood model

Further, we computed the power for the significant three-way interaction of image valence, image arousal, and music arousal in the memorisation likelihood model. The computation was specified in the same way as for the four-way interaction. Power was estimated at two sample sizes: our actual sample size (*n* = 41), resulting in 84.70% (95% CI [75.32–90.57%]) and a hypothetical *n* = 100 showing 99.00% (95% CI [94.55–99.97%]) power. Due to computational restraints, only 100 simulations were run. The confidence interval should therefore be interpreted cautiously, although the point estimate of the power is unlikely to change substantially with more simulations.

We computed the power analysis for the second significant three-way interaction of image valence, music valence and music arousal. We specified the power calculation the same way as for the other significant three-way interaction. Power was again estimated at two sample sizes: our actual sample size (*n* = 41), resulting in 87.00% (95% CI [78.80–92.89%]) and a hypothetical *n* = 100 showing 99.00% (95% CI [94.55–99.97%]) power.

## Discussion

This study investigated if and how image valence and arousal alone and their interaction with valence and arousal of simultaneously presented music excerpts influences memory for images. Additionally we examined the influence on memory discriminability and exploratorily investigated how this potential valence arousal interaction shapes basic visual attention.

### Memorisation likelihood (hit)

Hypothesis H1, expected high arousal images to be better memorised and discriminated, compared to low arousal images. Our results showed high memorisation likelihood for high arousal images, but not in all cases significantly different to low arousal images or across image valence. Our results reveal a more nuanced interaction with image valence and music arousal.

In low arousal music high arousal negative images were significantly better memorized compared to both positive and negative low arousal images. High arousal positive images do not significantly differ from both low arousal images and the high arousal negative images in memorisation likelihood. In this low arousal music context, this indicates that high arousal images trend towards higher memorisation likelihood compared to low arousal images, but that negative valence is needed for a decisive memory advantage over low arousal images.

In high arousal music context we observed that both positive and negative high arousal images were equally well remembered, but with insignificantly higher likelihood compared to low arousal negative images. However, low arousal positive images are significantly less likely to be remembered in high arousal music, compared to all other image types. Instead of attributing this to an advantage in memory for the low arousal negative images, we would rather frame it as a disadvantage for low arousal positive images. Low arousal positive images seem to fail to engage effective encoding mechanisms in the high arousal music context, which we would attribute to a lack of salience.

Overall our results carefully suggest, that in highly arousing music contexts images are memorised with similar likelihood, except for low arousal positive images. The lack of difference between the other image types, further suggests that in high arousal music context memorisation is not driven primarily by valence but by arousal, likely due to a global activation due to the high arousal music, indicating a vigilance mechanism. This heightened alertness promotes memory for objects, not strongly differentiating between image types. This is plausible, given that the consequences of missing a positive item would incur likely smaller consequences compared to missing out on a threat or negative item.

Taken together, the low-arousal music findings suggest that valence and arousal jointly shape memorisation. In contrast to the high-arousal music context, the low-arousal context leaves resources for valence-based memory candidate selection, showing a bias towards negative material.

### Memory discriminability (d’)

Concerning memory discriminability (d’), we found positive high-arousal music to significantly increase d’ compared to low arousal negative music. Contrary to expectation, we did not observe an effect of image arousal or valence nor any interaction of these. Although memorisation likelihood is partially influenced by an interaction of image valence and arousal, this interaction does not promote effective target/foil separation reflecting in the absence of image based effects in d’. The music context seems to have shaped the style and quality of encoding globally for all image types. We here assume, that in our experiment, positive arousing music has promoted broader attentional scope, providing more cues for retrieval by reducing confusion between similar items, leading to improved target/foil separation and hence higher overall d’.

### Fixation duration

Both average and total fixation durations significantly increased for high arousal images and decreased during high arousal music trials. The shortening of fixation durations under high-arousal music may reflect a vigilance mechanism. Here, arousing music likely promotes brief scanning of the visual scene to quickly obtain an overview. In this way, a visual scene can be scanned efficiently for potential threats or candidates for further disambiguation.

Our observation, that high arousal images produce longer fixations is in accordance with our hypothesis H2 and the literature, which suggests that high arousal stimuli can capture attention and sustain attention more easily, compared to low arousal items. Interestingly, this reflects partially in the memorisation likelihood for arousing images, but does not influence effective discrimination of memorised material. However, we did not find any significant effect of image or music valence on the fixation duration on an image per trial. The observed pattern suggests that certain images or music conditions influenced how long participants looked at items once fixated. Because the model for fixation counts showed severe diagnostic problems and could not be interpreted reliably, we cannot determine whether subtle effects on fixation frequency also existed. Therefore, our conclusions about visual attention must remain cautious: we can infer differences in fixation duration, but not differences in overall attentional investment across trials.

### Congruence

Our third hypothesis (H3) expected images which are valence congruent to the music, to be better memorised and discriminated, particularly in low-arousal music context, where lower arousal should leave more resources for valence based processing. We did not find clear indicators for valence congruence. Only one significant contrast in memorisation likelihood suggested such an effect, in which negative images in negative low arousal music are more likely remembered than positive images. In the absence of further consistent valence congruence patterns, we would frame this as an advantage of negative valence rather than a genuine congruence effect.

## Limitations

The main limitation in our study lies in sample size. While the sample size to detect medium effects for the three-way interactions was adequate, the four-way interaction between image and music valence and arousal was underpowered. Null effects should therefore be interpreted cautiously. However, for more reliable results, especially given the complexity of the models, a significantly larger sample size would be required.

## Conclusion

Overall we see memorisation mainly driven by high arousal and negative valence, while effects on visual attention appear primarily affected by arousal rather than valence. Negative material or context can suggest threat, and high arousal, especially in negative valence can signal a need for action or a need for change. In contrast, positive cues are likely lower in priority, as these potentially do not inflict negative consequences when missed. Visual attention is prolonged for high-arousal images, but shortened during high-arousal music. A valence agnostic vigilance mechanism in high arousal music contexts seems to promote memory across all stimulus types, as long as these are in some way engaging. However, these processes did not reflect in facilitated memory discrimination, with only the music context seemingly shaping encoding style and retrieval cues. In turn, only positive high arousal music promoted d’, likely due to the positive music affect eliciting broader processing style, with larger contextual focus, aiding retrieval. This summarises to a mainly arousal driven and negatively biased memory selection, with partial interactions of image valence and arousal, in specific contexts. Whilst high arousal activates a general memory and attention, low contextual arousal seems to allow for a more valence based appraisal.

## Supplemental Information

10.7717/peerj.20781/supp-1 Supplemental Information 1 Image stimuli with pre-test ratings Each image was rated in an online pre-test for valence and arousal on a 5-point Likert scale. The table shows the mean and standard deviation (SD) for each dimension, along with binary classifications used to categorize each image in the main experiment.

10.7717/peerj.20781/supp-2 Supplemental Information 2 Audio stimuli with pre-test ratings Each music excerpt was rated in an online pre-test for valence and arousal using a 5-point Likert scale. The table presents the mean and standard deviation (SD) for both dimensions, alongside binary classifications used to categorize each excerpt in the main experiment. The final column indicates the source of each music piece.

10.7717/peerj.20781/supp-3 Supplemental Information 3 Datasets and R code for analysis The ’Code’ folder contains an R markdown script and its html version (to view code without running R).
In the ’Data’ subfolder four datasets are provided: (1) a complete trial-level dataset with per-image data and all variables used in image-level analyses, (2) an aggregated dataset with per-trial variables used for trial-level models (e.g., d’), and (3–4) raw pretest ratings for the images and music pieces used in the experiment. The R Markdwon script loads the datasets automatically, when folder structure is as provided in this zip file. A Readme text file is included.

## Additional Information and Declarations

### Data Availability

The following information was supplied regarding data availability: The data and code are available in the Supplemental Files.
