# Supplementary material for: Valence–arousal interactions between images and music: differential effects on memorisation, discrimination, and fixations
Source: PeerJ. 2026 Apr 21;14:e20781. doi: 10.7717/peerj.20781 (PMC13108460; doi:10.7717/peerj.20781)
Supplement: Supplemental Information 1 — Each image was rated in an online pre-test for valence and arousal on a 5-point Likert scale. The table shows the mean and standard deviation (SD) for each dimension, along with binary classifications used to categorize each image in the main experiment. [file peerj-14-20781-s001.docx]

**Table S1**

*Image Stimuli with Pre-Test Ratings*

| Image Name | Database | Valence (mean) | Valence (SD) | Arousal (mean) | Arousal (SD) | Valence binary | Arousal binary |
| --- | --- | --- | --- | --- | --- | --- | --- |
| Acorns 1 | OASIS | 3.35 | 0.69 | 2.41 | 0.96 | 1 | 0 |
| Alcohol 8 | OASIS | 3.97 | 0.88 | 3.13 | 1.27 | 1 | 1 |
| Ambulance 2 | OASIS | 1.97 | 1.03 | 3.44 | 1.13 | 0 | 1 |
| Astronaut 1 | OASIS | 3.75 | 0.73 | 3.33 | 1.04 | 1 | 1 |
| Baby 6 | OASIS | 4.25 | 1.05 | 3.36 | 1.44 | 1 | 1 |
| Band 2 | OASIS | 3.69 | 0.72 | 3.42 | 0.84 | 1 | 1 |
| Bar 1 | OASIS | 3.25 | 0.94 | 2.81 | 1.01 | 1 | 0 |
| Beach 1 | OASIS | 4.53 | 0.74 | 3.47 | 1.47 | 1 | 1 |
| Bee 1 | OASIS | 3.29 | 0.82 | 2.87 | 1.06 | 1 | 0 |
| Billiards 1 | OASIS | 3.13 | 0.82 | 2.5 | 1.01 | 1 | 0 |
| Bird 3 | OASIS | 4.17 | 0.83 | 3.38 | 1.19 | 1 | 1 |
| Birthday 1 | OASIS | 4.12 | 0.98 | 3.5 | 1.05 | 1 | 1 |
| Bloody knife 1 | OASIS | 1.56 | 0.91 | 3.78 | 1.36 | 0 | 1 |
| Bored pose 2 | OASIS | 2.23 | 0.79 | 2.30 | 0.93 | 0 | 0 |
| Car 1 | OASIS | 3.38 | 0.94 | 3.42 | 0.99 | 1 | 1 |
| Cheerleader 1 | OASIS | 3.70 | 0.99 | 3.5 | 0.97 | 1 | 1 |
| Child labor 2 | OASIS | 1.96 | 0.94 | 2.96 | 0.98 | 0 | 0 |
| Chipmunk 3 | OASIS | 4 | 0.75 | 2.78 | 1.16 | 1 | 0 |
| Cockroach 1 | OASIS | 1.8 | 0.87 | 3.20 | 1.19 | 0 | 1 |
| Coffee 1 | OASIS | 4.12 | 0.85 | 3.45 | 1.08 | 1 | 1 |
| Couple 2 | OASIS | 4.04 | 0.72 | 3.56 | 1.02 | 1 | 1 |
| Cups 3 | OASIS | 3.35 | 0.66 | 2.38 | 0.98 | 1 | 0 |
| Destruction 2 | OASIS | 1.52 | 0.80 | 3.48 | 1.32 | 0 | 1 |
| Doctor 1 | OASIS | 2.73 | 0.78 | 2.70 | 0.84 | 0 | 0 |
| Dog 12 | OASIS | 4 | 0.99 | 3.42 | 1.06 | 1 | 1 |
| EM0002.jpg | EmoMadrid | 2.70 | 0.97 | 3.67 | 1.05 | 0 | 1 |
| EM0050.jpg | EmoMadrid | 3.70 | 0.84 | 3.5 | 0.94 | 1 | 1 |
| EM0053.jpg | EmoMadrid | 1.77 | 0.88 | 3.23 | 1.20 | 0 | 1 |
| EM0056.jpg | EmoMadrid | 3.83 | 0.93 | 3.31 | 1.00 | 1 | 1 |
| EM0075.jpg | EmoMadrid | 4.33 | 0.75 | 3.47 | 1.14 | 1 | 1 |
| EM0130.jpg | EmoMadrid | 3.41 | 1.09 | 3.20 | 1.19 | 1 | 1 |
| EM0140.jpg | EmoMadrid | 2.30 | 0.99 | 3.13 | 1.22 | 0 | 1 |
| EM0153.jpg | EmoMadrid | 4.14 | 0.76 | 3.14 | 1.31 | 1 | 1 |
| EM0176.jpg | EmoMadrid | 4.30 | 0.74 | 2.97 | 1.28 | 1 | 0 |
| EM0311.jpg | EmoMadrid | 3.92 | 0.94 | 3.44 | 1.16 | 1 | 1 |
| EM0381.jpg | EmoMadrid | 3.94 | 0.76 | 2.74 | 1.04 | 1 | 0 |
| EM0400.jpg | EmoMadrid | 1.76 | 1.02 | 3.32 | 1.47 | 0 | 1 |
| EM0496.jpg | EmoMadrid | 3.77 | 0.82 | 3.12 | 1.11 | 1 | 1 |
| EM0556.jpg | EmoMadrid | 4 | 0.74 | 2.58 | 1.10 | 1 | 0 |
| EM0621.jpg | EmoMadrid | 1.69 | 1.04 | 2.79 | 1.11 | 0 | 0 |
| EM0710.jpg | EmoMadrid | 1.46 | 0.76 | 3.65 | 1.38 | 0 | 1 |
| EM0741.jpg | EmoMadrid | 1.48 | 0.69 | 3.69 | 1.20 | 0 | 1 |
| EM0746.jpg | EmoMadrid | 4.21 | 0.74 | 3.32 | 1.04 | 1 | 1 |
| EM0949.jpg | EmoMadrid | 4.08 | 0.70 | 3.38 | 0.96 | 1 | 1 |
| EM1057.jpg | EmoMadrid | 1.68 | 1.04 | 3.16 | 1.22 | 0 | 1 |
| EM_0818.jpg | EmoMadrid | 1.61 | 0.89 | 3.87 | 1.14 | 0 | 1 |
| Explosion 1 | OASIS | 2.11 | 1.01 | 3.55 | 1.08 | 0 | 1 |
| Fence 4 | OASIS | 1.75 | 0.73 | 2.72 | 1.11 | 0 | 0 |
| Fireman 1 | OASIS | 2.23 | 0.88 | 3.23 | 1.02 | 0 | 1 |
| Fireworks 1 | OASIS | 3.91 | 1.09 | 3.77 | 1.00 | 1 | 1 |
| Flood 1 | OASIS | 1.63 | 0.74 | 3.11 | 1.22 | 0 | 1 |
| Frisbee 1 | OASIS | 3.83 | 0.83 | 3.38 | 1.00 | 1 | 1 |
| Galaxy 1 | OASIS | 3.46 | 1 | 3.51 | 1.05 | 1 | 1 |
| Garbage dump 3 | OASIS | 1.67 | 0.62 | 2.85 | 1.03 | 0 | 0 |
| Grass 2 | OASIS | 3.61 | 0.90 | 2.67 | 1.24 | 1 | 0 |
| Gun 10 | OASIS | 1.69 | 0.81 | 3.34 | 1.37 | 0 | 1 |
| Hangover 1 | OASIS | 1.86 | 0.76 | 2.83 | 1.3 | 0 | 0 |
| Horse 1 | OASIS | 4.03 | 0.76 | 2.88 | 1.04 | 1 | 0 |
| Lake 3 | OASIS | 4.59 | 0.70 | 3.44 | 1.24 | 1 | 1 |
| Lightning 2 | OASIS | 2.90 | 0.87 | 3.68 | 0.79 | 0 | 1 |
| Meerkat 1 | OASIS | 3.61 | 1.01 | 3.31 | 1.05 | 1 | 1 |
| Monkey 3 | OASIS | 2.66 | 0.97 | 2.94 | 1.14 | 0 | 0 |
| Mother 3 | OASIS | 3.62 | 0.97 | 3.35 | 1.02 | 1 | 1 |
| Opossum 1 | OASIS | 2.70 | 1.10 | 3.46 | 0.93 | 0 | 1 |
| Orangutan 1 | OASIS | 3.57 | 0.94 | 2.86 | 0.99 | 1 | 0 |
| Penguins 2 | OASIS | 4.29 | 0.63 | 3.29 | 1.03 | 1 | 1 |
| Police 1 | OASIS | 2.19 | 0.79 | 3.39 | 0.92 | 0 | 1 |
| Power lines 1 | OASIS | 2.81 | 0.69 | 2.59 | 0.95 | 0 | 0 |
| Raccoon | OASIS | 2.55 | 1.19 | 3.39 | 1.10 | 0 | 1 |
| Rooster 1 | OASIS | 2.74 | 0.74 | 2.83 | 0.86 | 0 | 0 |
| Skyscraper 1 | OASIS | 3.07 | 0.72 | 3.07 | 1.07 | 1 | 0 |
| Tornado 4 | OASIS | 2 | 0.74 | 3.57 | 1.04 | 0 | 1 |
| Wedding 7 | OASIS | 3.57 | 0.72 | 2.81 | 1.04 | 1 | 0 |
| Wolf 2 | OASIS | 2.40 | 0.90 | 3.65 | 0.89 | 0 | 1 |
