# Supplementary material for: Valence–arousal interactions between images and music: differential effects on memorisation, discrimination, and fixations
Source: PeerJ. 2026 Apr 21;14:e20781. doi: 10.7717/peerj.20781 (PMC13108460; doi:10.7717/peerj.20781)
Supplement: Supplemental Information 2 — Each music excerpt was rated in an online pre-test for valence and arousal using a 5-point Likert scale. The table presents the mean and standard deviation (SD) for both dimensions, alongside binary classifications used to categorize each excerpt in the main experiment. The final column indicates the source of each music piece. [file peerj-14-20781-s002.docx]

**Table S2**

*Audio Stimuli with Pre-Test Ratings*

| Music | Valence (mean) | Valence (SD) | Arousal (mean) | Arousal (SD) | Valence binary | Arousal binary | Source |
| --- | --- | --- | --- | --- | --- | --- | --- |
| Batman Returns Tr. 5 | 1.81 | 0.73 | 3.55 | 1.22 | 0 | 1 | Eerola & Vuoskoski (2011) |
| OnTheWay 0:00 - 0:15 | 3.57 | 1.02 | 2.67 | 1.15 | 1 | 0 | Shockwave Sound |
| Divertimento in D Maj. K. 136: III. Presto (1:10 - 1:25) | 3.98 | 0.78 | 3.80 | 0.73 | 1 | 1 | Mozart |
| On the Nature of Daylight 0:00 - 0:15 | 2.63 | 1.15 | 2.63 | 1.2 | 0 | 0 | Dinah Washington |
| Buddha Bar 0:00 - 0:15 | 3.49 | 0.94 | 2.59 | 1.17 | 1 | 0 | Buddha Bar |
| Man of Galilee CD 1 - Tr 2 | 3.61 | 0.69 | 3.53 | 0.81 | 1 | 1 | Eerola & Vuoskoski (2011) |
| AlienTrilogy Tr. 5 | 1.8 | 0.84 | 3.71 | 1.19 | 0 | 1 | Eerola & Vuoskoski (2011) |
| The English Patient Tr. 18 | 2.84 | 0.97 | 2.79 | 1 | 0 | 0 | Eerola & Vuoskoski (2011) |
