# Supplementary material for: Valence–arousal interactions between images and music: differential effects on memorisation, discrimination, and fixations
Source: PeerJ. 2026 Apr 21;14:e20781. doi: 10.7717/peerj.20781 (PMC13108460; doi:10.7717/peerj.20781)
Supplement: Supplemental Information 3 — The ’Code’ folder contains an R markdown script and its html version (to view code without running R). In the ’Data’ subfolder four datasets are provided: (1) a complete trial-level dataset with per-image data and all variables used in image-level analyses, (2) an aggregated dataset with per-trial variables used for trial-level models (e.g., d’), and (3–4) raw pretest ratings for the images and music pieces used in the experiment. The R Markdwon script loads the datasets automatically, when folder structure is as provided in this zip file. A Readme text file is included. [file peerj-14-20781-s003.zip › Analysis_R_code_Revision/Code/Analysis_Code_revision.html]

rcode\_revision


# rcode\_revision

#### DDB

# #

# REVISION

# #

The revision results are based on the code below

#### Load Libraries

```
# Not all libraries are used
library(brms)
```

```
## Warning: Paket 'brms' wurde unter R Version 4.2.3 erstellt
```

```
## Lade nötiges Paket: Rcpp
```

```
## Loading 'brms' package (version 2.20.4). Useful instructions
## can be found by typing help('brms'). A more detailed introduction
## to the package is available through vignette('brms_overview').
```

```
## 
## Attache Paket: 'brms'
```

```
## Das folgende Objekt ist maskiert 'package:stats':
## 
##     ar
```

```
library(car)
```

```
## Lade nötiges Paket: carData
```

```
library(DHARMa)
```

```
## This is DHARMa 0.4.7. For overview type '?DHARMa'. For recent changes, type news(package = 'DHARMa')
```

```
library(dplyr)
```

```
## Warning: Paket 'dplyr' wurde unter R Version 4.2.3 erstellt
```

```
## 
## Attache Paket: 'dplyr'
```

```
## Das folgende Objekt ist maskiert 'package:car':
## 
##     recode
```

```
## Die folgenden Objekte sind maskiert von 'package:stats':
## 
##     filter, lag
```

```
## Die folgenden Objekte sind maskiert von 'package:base':
## 
##     intersect, setdiff, setequal, union
```

```
library(e1071)
```

```
## Warning: Paket 'e1071' wurde unter R Version 4.2.3 erstellt
```

```
## 
## Attache Paket: 'e1071'
```

```
## Das folgende Objekt ist maskiert 'package:brms':
## 
##     rwiener
```

```
library(easystats)
```

```
## Warning: Paket 'easystats' wurde unter R Version 4.2.3 erstellt
```

```
## # Attaching packages: easystats 0.7.0 (red = needs update)
## ✖ bayestestR  0.13.1   ✖ correlation 0.8.4 
## ✖ datawizard  0.9.0    ✖ effectsize  0.8.6 
## ✖ insight     0.19.7   ✖ modelbased  0.8.6 
## ✖ performance 0.10.8   ✖ parameters  0.21.3
## ✖ report      0.5.8    ✖ see         0.8.1 
## 
## Restart the R-Session and update packages with `easystats::easystats_update()`.
```

```
library(effsize)
```

```
## Warning: Paket 'effsize' wurde unter R Version 4.2.3 erstellt
```

```
library(emmeans)
library(ggplot2)
```

```
## Warning: Paket 'ggplot2' wurde unter R Version 4.2.3 erstellt
```

```
library(ggpubr)
```

```
## Warning: Paket 'ggpubr' wurde unter R Version 4.2.3 erstellt
```

```
## 
## Attache Paket: 'ggpubr'
```

```
## Die folgenden Objekte sind maskiert von 'package:datawizard':
## 
##     mean_sd, median_mad
```

```
library(here)
library(jtools)
```

```
## Warning: Paket 'jtools' wurde unter R Version 4.2.3 erstellt
```

```
## 
## Attache Paket: 'jtools'
```

```
## Das folgende Objekt ist maskiert 'package:modelbased':
## 
##     standardize
```

```
## Die folgenden Objekte sind maskiert von 'package:insight':
## 
##     get_data, get_weights
```

```
## Das folgende Objekt ist maskiert 'package:effectsize':
## 
##     standardize
```

```
## Die folgenden Objekte sind maskiert von 'package:datawizard':
## 
##     center, standardize
```

```
library(lmtest)
```

```
## Warning: Paket 'lmtest' wurde unter R Version 4.2.3 erstellt
```

```
## Lade nötiges Paket: zoo
```

```
## Warning: Paket 'zoo' wurde unter R Version 4.2.3 erstellt
```

```
## 
## Attache Paket: 'zoo'
```

```
## Die folgenden Objekte sind maskiert von 'package:base':
## 
##     as.Date, as.Date.numeric
```

```
library(lme4)
```

```
## Warning: Paket 'lme4' wurde unter R Version 4.2.3 erstellt
```

```
## Lade nötiges Paket: Matrix
```

```
## Warning: Paket 'Matrix' wurde unter R Version 4.2.3 erstellt
```

```
## 
## Attache Paket: 'lme4'
```

```
## Das folgende Objekt ist maskiert 'package:brms':
## 
##     ngrps
```

```
library(lmeresampler)
```

```
## Warning: Paket 'lmeresampler' wurde unter R Version 4.2.3 erstellt
```

```
library(lmerTest)
```

```
## Warning: Paket 'lmerTest' wurde unter R Version 4.2.3 erstellt
```

```
## 
## Attache Paket: 'lmerTest'
```

```
## Das folgende Objekt ist maskiert 'package:lme4':
## 
##     lmer
```

```
## Das folgende Objekt ist maskiert 'package:stats':
## 
##     step
```

```
library(MuMIn)
```

```
## 
## Attache Paket: 'MuMIn'
```

```
## Das folgende Objekt ist maskiert 'package:insight':
## 
##     get_call
```

```
## Das folgende Objekt ist maskiert 'package:brms':
## 
##     loo
```

```
library(nlme)
```

```
## 
## Attache Paket: 'nlme'
```

```
## Das folgende Objekt ist maskiert 'package:lme4':
## 
##     lmList
```

```
## Das folgende Objekt ist maskiert 'package:dplyr':
## 
##     collapse
```

```
library(performance)
library(randtests)
```

```
## Warning: Paket 'randtests' wurde unter R Version 4.2.3 erstellt
```

```
library(report)
library(simr)
```

```
## Warning: Paket 'simr' wurde unter R Version 4.2.3 erstellt
```

```
## 
## Attache Paket: 'simr'
```

```
## Die folgenden Objekte sind maskiert von 'package:nlme':
## 
##     coef<-, getData
```

```
## Das folgende Objekt ist maskiert 'package:lme4':
## 
##     getData
```

#### Load data

```
# Complete Dataset on the image level
complete_data <- read.csv('Data/complete_data_image_level_r.csv', sep=';')
# Aggregated dataset on the trial level
data_agg2 <- read.csv('Data/data_complete_aggregated_r_v2.csv', sep=';')
# Same as aggregated data, but includes image valence and image arousal

# Data from the image rating pre-test
pretest_img_rating <- read.csv('Data/pre-test_image_ratings_final.csv', sep=';')
# Data from the music rating pre-test
pretest_mus_rating <- read.csv('Data/pre-test_music_ratings_final.csv', sep=';')


### Create Subsets
# Data Looked At Images Only
complete_data_looked_at <- subset(complete_data, Number_of_fix_to_image_per_trial >=1) 

# Data looked at target
complete_data_looked_at_target <- subset(complete_data_looked_at, Is_Target_Sel = "Target")

# Data looked at exnoaudio 
complete_data_looked_at_exnoaudio <- subset(complete_data_looked_at, MusicAffect != "noaudio")

# Data looked at exnoaudio target
complete_data_looked_at_exnoaudio_target <- subset(complete_data_looked_at_exnoaudio, Is_Target_Sel = "Target")

# Data looked at exnoaudio (duplicate assignment kept in place)
CompLookedExNoAudio <- complete_data_looked_at_exnoaudio

# Data distractor
complete_data_distractor <- subset(complete_data, Is_Target_Sel == "Distractor")

# Create a subset of only target images present during the presentation phase (i.e. target images in selection phase are the ones shown before. Distractors would be the new images not shown in the presentation phase) "Sel" later on in the script refers to images from the selection phase, whilst "Pres" refers to images from the presentation phase.
# Data target

complete_data_target <- subset(complete_data, Is_Target_Sel == "Target")
```

#### Create Music Valence Music Arousal Variables

```
CompLookedExNoAudio$val_mus <- ifelse(CompLookedExNoAudio$MusicAffect %in% c("happy", "tender"), 1, 0)  # positive valence
CompLookedExNoAudio$arous_mus <- ifelse(CompLookedExNoAudio$MusicAffect %in% c("happy", "fear"), 1, 0)  # high arousal

# Check mapping
unique(CompLookedExNoAudio[, c("MusicAffect", "val_mus", "arous_mus")])
```

```
##    MusicAffect val_mus arous_mus
## 2         fear       0         1
## 13      tender       1         0
## 25       happy       1         1
## 37         sad       0         0
```

## Model Hit ~ Img V + Img A + Mus V + Mus A

### Make Variables Factors

```
CompLookedExNoAudio$Valence_Sel <- factor(CompLookedExNoAudio$Valence_Sel,
                                          levels = c(0,1))
CompLookedExNoAudio$Arousal_Sel <- factor(CompLookedExNoAudio$Arousal_Sel,
                                          levels = c(0,1))
CompLookedExNoAudio$val_mus <- factor(CompLookedExNoAudio$val_mus,
                                      levels = c(0,1))
CompLookedExNoAudio$arous_mus <- factor(CompLookedExNoAudio$arous_mus,
                                        levels = c(0,1))
```

### Create effect coded Var

```
# Create effect-coded versions of each predictor
CompLookedExNoAudio$Valence_Sel_fx <- ifelse(CompLookedExNoAudio$Valence_Sel == 1, 0.5, -0.5)
CompLookedExNoAudio$Arousal_Sel_fx <- ifelse(CompLookedExNoAudio$Arousal_Sel == 1, 0.5, -0.5)
CompLookedExNoAudio$val_mus_fx     <- ifelse(CompLookedExNoAudio$val_mus     == 1, 0.5, -0.5)
CompLookedExNoAudio$arous_mus_fx   <- ifelse(CompLookedExNoAudio$arous_mus   == 1, 0.5, -0.5)
```

### Model Effect Coded

```
# Effect coded
model_hit_img_mus_VA_fx <- glmer(
  Hit ~ Valence_Sel_fx * Arousal_Sel_fx * val_mus_fx * arous_mus_fx + mean_pupil_per_trial +
    (1 | Subject),
  data = CompLookedExNoAudio,
  family = binomial(link = "logit"),
  control = glmerControl(optimizer = "bobyqa",
                         optCtrl = list(maxfun = 200000)))
```

#### Summary

```
summary(model_hit_img_mus_VA_fx)
```

```
## Generalized linear mixed model fit by maximum likelihood (Laplace
##   Approximation) [glmerMod]
##  Family: binomial  ( logit )
## Formula: Hit ~ Valence_Sel_fx * Arousal_Sel_fx * val_mus_fx * arous_mus_fx +  
##     mean_pupil_per_trial + (1 | Subject)
##    Data: CompLookedExNoAudio
## Control: glmerControl(optimizer = "bobyqa", optCtrl = list(maxfun = 2e+05))
## 
##      AIC      BIC   logLik deviance df.resid 
##  14268.7  14401.1  -7116.3  14232.7    11567 
## 
## Scaled residuals: 
##     Min      1Q  Median      3Q     Max 
## -0.9577 -0.7041 -0.6056  1.2765  2.4421 
## 
## Random effects:
##  Groups  Name        Variance Std.Dev.
##  Subject (Intercept) 0.06981  0.2642  
## Number of obs: 11585, groups:  Subject, 41
## 
## Fixed effects:
##                                                       Estimate Std. Error
## (Intercept)                                           -0.80126    0.04682
## Valence_Sel_fx                                        -0.12495    0.04060
## Arousal_Sel_fx                                         0.25322    0.04061
## val_mus_fx                                             0.02570    0.04111
## arous_mus_fx                                          -0.03053    0.04130
## mean_pupil_per_trial                                  -0.01252    0.11767
## Valence_Sel_fx:Arousal_Sel_fx                          0.05256    0.08120
## Valence_Sel_fx:val_mus_fx                              0.18569    0.08120
## Arousal_Sel_fx:val_mus_fx                              0.04094    0.08120
## Valence_Sel_fx:arous_mus_fx                           -0.06309    0.08120
## Arousal_Sel_fx:arous_mus_fx                            0.02467    0.08121
## val_mus_fx:arous_mus_fx                                0.14318    0.08322
## Valence_Sel_fx:Arousal_Sel_fx:val_mus_fx              -0.12954    0.16239
## Valence_Sel_fx:Arousal_Sel_fx:arous_mus_fx             0.52711    0.16241
## Valence_Sel_fx:val_mus_fx:arous_mus_fx                -0.33253    0.16239
## Arousal_Sel_fx:val_mus_fx:arous_mus_fx                -0.22542    0.16242
## Valence_Sel_fx:Arousal_Sel_fx:val_mus_fx:arous_mus_fx -0.09029    0.32478
##                                                       z value Pr(>|z|)    
## (Intercept)                                           -17.113  < 2e-16 ***
## Valence_Sel_fx                                         -3.078  0.00209 ** 
## Arousal_Sel_fx                                          6.236 4.49e-10 ***
## val_mus_fx                                              0.625  0.53192    
## arous_mus_fx                                           -0.739  0.45980    
## mean_pupil_per_trial                                   -0.106  0.91526    
## Valence_Sel_fx:Arousal_Sel_fx                           0.647  0.51749    
## Valence_Sel_fx:val_mus_fx                               2.287  0.02220 *  
## Arousal_Sel_fx:val_mus_fx                               0.504  0.61413    
## Valence_Sel_fx:arous_mus_fx                            -0.777  0.43718    
## Arousal_Sel_fx:arous_mus_fx                             0.304  0.76127    
## val_mus_fx:arous_mus_fx                                 1.720  0.08535 .  
## Valence_Sel_fx:Arousal_Sel_fx:val_mus_fx               -0.798  0.42503    
## Valence_Sel_fx:Arousal_Sel_fx:arous_mus_fx              3.246  0.00117 ** 
## Valence_Sel_fx:val_mus_fx:arous_mus_fx                 -2.048  0.04059 *  
## Arousal_Sel_fx:val_mus_fx:arous_mus_fx                 -1.388  0.16519    
## Valence_Sel_fx:Arousal_Sel_fx:val_mus_fx:arous_mus_fx  -0.278  0.78100    
## ---
## Signif. codes:  0 '***' 0.001 '**' 0.01 '*' 0.05 '.' 0.1 ' ' 1
```

```
## 
## Correlation matrix not shown by default, as p = 17 > 12.
## Use print(x, correlation=TRUE)  or
##     vcov(x)        if you need it
```

#### Anova

```
Anova(model_hit_img_mus_VA_fx, type = 2)
```

```
## Analysis of Deviance Table (Type II Wald chisquare tests)
## 
## Response: Hit
##                                                         Chisq Df Pr(>Chisq)    
## Valence_Sel_fx                                         9.0317  1   0.002653 ** 
## Arousal_Sel_fx                                        37.8994  1  7.449e-10 ***
## val_mus_fx                                             0.3694  1   0.543305    
## arous_mus_fx                                           0.4818  1   0.487593    
## mean_pupil_per_trial                                   0.0113  1   0.915263    
## Valence_Sel_fx:Arousal_Sel_fx                          0.4126  1   0.520646    
## Valence_Sel_fx:val_mus_fx                              4.9766  1   0.025692 *  
## Arousal_Sel_fx:val_mus_fx                              0.2699  1   0.603370    
## Valence_Sel_fx:arous_mus_fx                            0.4420  1   0.506144    
## Arousal_Sel_fx:arous_mus_fx                            0.0323  1   0.857374    
## val_mus_fx:arous_mus_fx                                2.9001  1   0.088574 .  
## Valence_Sel_fx:Arousal_Sel_fx:val_mus_fx               0.6390  1   0.424079    
## Valence_Sel_fx:Arousal_Sel_fx:arous_mus_fx            10.4920  1   0.001199 ** 
## Valence_Sel_fx:val_mus_fx:arous_mus_fx                 4.2655  1   0.038894 *  
## Arousal_Sel_fx:val_mus_fx:arous_mus_fx                 1.9074  1   0.167249    
## Valence_Sel_fx:Arousal_Sel_fx:val_mus_fx:arous_mus_fx  0.0773  1   0.781000    
## ---
## Signif. codes:  0 '***' 0.001 '**' 0.01 '*' 0.05 '.' 0.1 ' ' 1
```

##### ICC

```
icc(model_hit_img_mus_VA_fx)
```

```
## # Intraclass Correlation Coefficient
## 
##     Adjusted ICC: 0.021
##   Unadjusted ICC: 0.021
```

```
# Adjusted prop variance explained by random effects after accounting for fixed fx (unadjusted: without accounting for fixed fx) We use adjusted
```

##### R2

```
r2(model_hit_img_mus_VA_fx) # Marginal = fixed FX
```

```
## # R2 for Mixed Models
## 
##   Conditional R2: 0.030
##      Marginal R2: 0.009
```

```
                            # Conditional fixed FX + random FX
```

### Diagnostics

#### Check Multicollinearity

```
library(performance)
check_collinearity(model_hit_img_mus_VA_fx)
```

```
## # Check for Multicollinearity
## 
## Low Correlation
## 
##                                                   Term  VIF   VIF 95% CI
##                                         Valence_Sel_fx 1.01 [1.00, 1.12]
##                                         Arousal_Sel_fx 1.00 [1.00, 1.35]
##                                             val_mus_fx 1.01 [1.00, 1.10]
##                                           arous_mus_fx 1.01 [1.00, 1.08]
##                                   mean_pupil_per_trial 1.00 [1.00, 2.34]
##                          Valence_Sel_fx:Arousal_Sel_fx 1.01 [1.00, 1.10]
##                              Valence_Sel_fx:val_mus_fx 1.01 [1.00, 1.11]
##                              Arousal_Sel_fx:val_mus_fx 1.01 [1.00, 1.10]
##                            Valence_Sel_fx:arous_mus_fx 1.01 [1.00, 1.11]
##                            Arousal_Sel_fx:arous_mus_fx 1.01 [1.00, 1.11]
##                                val_mus_fx:arous_mus_fx 1.01 [1.00, 1.10]
##               Valence_Sel_fx:Arousal_Sel_fx:val_mus_fx 1.01 [1.00, 1.10]
##             Valence_Sel_fx:Arousal_Sel_fx:arous_mus_fx 1.01 [1.00, 1.13]
##                 Valence_Sel_fx:val_mus_fx:arous_mus_fx 1.01 [1.00, 1.11]
##                 Arousal_Sel_fx:val_mus_fx:arous_mus_fx 1.01 [1.00, 1.11]
##  Valence_Sel_fx:Arousal_Sel_fx:val_mus_fx:arous_mus_fx 1.01 [1.00, 1.10]
##  Increased SE Tolerance Tolerance 95% CI
##          1.00      0.99     [0.89, 1.00]
##          1.00      1.00     [0.74, 1.00]
##          1.00      0.99     [0.91, 1.00]
##          1.00      0.99     [0.93, 1.00]
##          1.00      1.00     [0.43, 1.00]
##          1.00      0.99     [0.91, 1.00]
##          1.00      0.99     [0.90, 1.00]
##          1.00      0.99     [0.91, 1.00]
##          1.00      0.99     [0.90, 1.00]
##          1.00      0.99     [0.90, 1.00]
##          1.00      0.99     [0.91, 1.00]
##          1.00      0.99     [0.91, 1.00]
##          1.00      0.99     [0.88, 1.00]
##          1.00      0.99     [0.90, 1.00]
##          1.00      0.99     [0.90, 1.00]
##          1.00      0.99     [0.91, 1.00]
```

#### Simulate Residuals

```
library(DHARMa)

# simulate standardized residuals (store with _hit suffix)
sim_res_hit <- simulateResiduals(fittedModel = model_hit_img_mus_VA_fx, n = 1000)
plot(sim_res_hit)   # default DHARMa plot: QQ, residuals vs predicted, histogram, etc.
```

#### Uniformity Test

```
uni_test_hit <- testUniformity(sim_res_hit)
```

```
uni_test_hit
```

```
## 
##  Asymptotic one-sample Kolmogorov-Smirnov test
## 
## data:  simulationOutput$scaledResiduals
## D = 0.0073629, p-value = 0.5564
## alternative hypothesis: two-sided
```

#### Dispersion Test

```
disp_test_hit <- testDispersion(sim_res_hit)
```

```
disp_test_hit
```

```
## 
##  DHARMa nonparametric dispersion test via sd of residuals fitted vs.
##  simulated
## 
## data:  simulationOutput
## dispersion = 1.0001, p-value = 0.97
## alternative hypothesis: two.sided
```

#### Outlier Test

```
outlier_test_hit <- testOutliers(sim_res_hit)
```

```
outlier_test_hit
```

```
## 
##  DHARMa outlier test based on exact binomial test with approximate
##  expectations
## 
## data:  sim_res_hit
## outliers at both margin(s) = 21, observations = 11585, p-value = 0.7547
## alternative hypothesis: true probability of success is not equal to 0.001998002
## 95 percent confidence interval:
##  0.001122422 0.002769560
## sample estimates:
## frequency of outliers (expected: 0.001998001998002 ) 
##                                          0.001812689
```

#### Zero Inflation Test

```
zi_test_hit <- testZeroInflation(sim_res_hit)
```

```
zi_test_hit
```

```
## 
##  DHARMa zero-inflation test via comparison to expected zeros with
##  simulation under H0 = fitted model
## 
## data:  simulationOutput
## ratioObsSim = 1.0002, p-value = 0.964
## alternative hypothesis: two.sided
```

### Emmeans Valence\_Sel × Arousal\_Sel × arous\_mus

```
# 1. Valence_Sel × Arousal_Sel × arous_mus
emm1 <- emmeans(model_hit_img_mus_VA_fx, 
                ~ Valence_Sel_fx * Arousal_Sel_fx * arous_mus_fx, 
                type = "response", 
                infer = c(TRUE, TRUE))  # adds lower.CL and upper.CL
```

```
## NOTE: Results may be misleading due to involvement in interactions
```

```
# Standard Plot
# plot_contr_vI_AI_AM <- plot(emm1,comparisons = TRUE)
  

contr1 <- contrast(emm1, method = "pairwise", adjust = "tukey", infer = c(TRUE, TRUE)) %>%
  as.data.frame() %>%
  filter(p.value < 0.05)

contr1
```

```
##  contrast                                                                                                          
##  (Valence_Sel_fx-0.5 Arousal_Sel_fx-0.5 arous_mus_fx-0.5) / (Valence_Sel_fx-0.5 Arousal_Sel_fx0.5 arous_mus_fx-0.5)
##  (Valence_Sel_fx0.5 Arousal_Sel_fx-0.5 arous_mus_fx-0.5) / (Valence_Sel_fx-0.5 Arousal_Sel_fx0.5 arous_mus_fx-0.5) 
##  (Valence_Sel_fx-0.5 Arousal_Sel_fx0.5 arous_mus_fx-0.5) / (Valence_Sel_fx0.5 Arousal_Sel_fx-0.5 arous_mus_fx0.5)  
##  (Valence_Sel_fx0.5 Arousal_Sel_fx0.5 arous_mus_fx-0.5) / (Valence_Sel_fx0.5 Arousal_Sel_fx-0.5 arous_mus_fx0.5)   
##  (Valence_Sel_fx-0.5 Arousal_Sel_fx-0.5 arous_mus_fx0.5) / (Valence_Sel_fx0.5 Arousal_Sel_fx-0.5 arous_mus_fx0.5)  
##  (Valence_Sel_fx0.5 Arousal_Sel_fx-0.5 arous_mus_fx0.5) / (Valence_Sel_fx-0.5 Arousal_Sel_fx0.5 arous_mus_fx0.5)   
##  (Valence_Sel_fx0.5 Arousal_Sel_fx-0.5 arous_mus_fx0.5) / Valence_Sel_fx0.5 Arousal_Sel_fx0.5 arous_mus_fx0.5      
##  odds.ratio     SE  df asymp.LCL asymp.UCL null z.ratio p.value
##       0.707 0.0572 Inf     0.553     0.904    1  -4.280  0.0005
##       0.716 0.0574 Inf     0.561     0.913    1  -4.169  0.0008
##       1.717 0.1416 Inf     1.337     2.205    1   6.551  <.0001
##       1.407 0.1185 Inf     1.090     1.816    1   4.055  0.0013
##       1.370 0.1143 Inf     1.064     1.764    1   3.770  0.0041
##       0.656 0.0540 Inf     0.511     0.842    1  -5.127  <.0001
##       0.655 0.0540 Inf     0.510     0.841    1  -5.132  <.0001
## 
## Results are averaged over the levels of: val_mus_fx 
## Confidence level used: 0.95 
## Conf-level adjustment: tukey method for comparing a family of 8 estimates 
## Intervals are back-transformed from the log odds ratio scale 
## P value adjustment: tukey method for comparing a family of 8 estimates 
## Tests are performed on the log odds ratio scale
```

```
###
# 1. Update factor levels in the emmGrid
# Convert effect-coded levels to readable labels
emm1@grid$Valence_Sel_fx <- factor(ifelse(emm1@grid$Valence_Sel_fx == 0.5, "Pos", "Neg"))
emm1@grid$Arousal_Sel_fx <- factor(ifelse(emm1@grid$Arousal_Sel_fx == 0.5, "High", "Low"))
emm1@grid$arous_mus_fx   <- factor(ifelse(emm1@grid$arous_mus_fx == 0.5, "High", "Low"))

# Plot
plot_contr_vI_AI_AM <- plot(emm1, type = "response", comparisons = TRUE)


# 2. continue with original plotting code
plot_contr_vI_AI_AM <- plot(emm1, type = "response", comparisons = TRUE)

# Change comparison arrows (layers 3 and 4) to black/grey and dotted lines
plot_contr_vI_AI_AM$layers[[3]]$aes_params$colour <- "grey30"
plot_contr_vI_AI_AM$layers[[3]]$aes_params$linetype <- "dotted"
plot_contr_vI_AI_AM$layers[[4]]$aes_params$colour <- "grey30"
plot_contr_vI_AI_AM$layers[[4]]$aes_params$linetype <- "dotted"

# Change main CI and point colors to black
plot_contr_vI_AI_AM$layers[[1]]$aes_params$colour <- "black"
plot_contr_vI_AI_AM$layers[[2]]$aes_params$colour <- "black"

# Apply theme and labels
plot_contr_vI_AI_AM_modified <- plot_contr_vI_AI_AM +
  theme_apa() +
  theme(
    panel.border = element_blank(),
    axis.line = element_line(color = "black"),
    axis.line.y.right = element_blank(),
    axis.line.x.top = element_blank()
  ) +
  labs(
    x = "Probability of correct recognition (Hit)",
    y = "Valence Img. x Arousal Img. x Arousal Mus."
  )

# Print updated plot
plot_contr_vI_AI_AM_modified
```

### Emmeans Valence\_Sel × val\_mus × arous\_mus

```
# 2. Valence_Sel × val_mus × arous_mus
emm2 <- emmeans(model_hit_img_mus_VA_fx, 
                ~ Valence_Sel_fx * val_mus_fx * arous_mus_fx, 
                type = "response", 
                infer = c(TRUE, TRUE))
```

```
## NOTE: Results may be misleading due to involvement in interactions
```

```
plot(emm2,comparisons = TRUE)
```

```
contr2 <- contrast(emm2, method = "pairwise",adjust = "tukey", infer = c(TRUE, TRUE)) %>%
  as.data.frame() %>%
  filter(p.value < 0.05)
contr2
```

```
##  contrast                                                                                                  
##  (Valence_Sel_fx-0.5 val_mus_fx-0.5 arous_mus_fx-0.5) / (Valence_Sel_fx0.5 val_mus_fx-0.5 arous_mus_fx-0.5)
##  (Valence_Sel_fx-0.5 val_mus_fx-0.5 arous_mus_fx-0.5) / (Valence_Sel_fx0.5 val_mus_fx-0.5 arous_mus_fx0.5) 
##  (Valence_Sel_fx0.5 val_mus_fx-0.5 arous_mus_fx0.5) / (Valence_Sel_fx-0.5 val_mus_fx0.5 arous_mus_fx0.5)   
##  odds.ratio     SE  df asymp.LCL asymp.UCL null z.ratio p.value
##       1.309 0.1069 Inf     1.022     1.677    1   3.299  0.0218
##       1.377 0.1146 Inf     1.070     1.772    1   3.843  0.0031
##       0.776 0.0636 Inf     0.605     0.995    1  -3.094  0.0415
## 
## Results are averaged over the levels of: Arousal_Sel_fx 
## Confidence level used: 0.95 
## Conf-level adjustment: tukey method for comparing a family of 8 estimates 
## Intervals are back-transformed from the log odds ratio scale 
## P value adjustment: tukey method for comparing a family of 8 estimates 
## Tests are performed on the log odds ratio scale
```

```
# APA Style Plot
library(emmeans)
library(ggplot2)
library(apaTables)
```

```
## Warning: Paket 'apaTables' wurde unter R Version 4.2.3 erstellt
```

```
library(here)
library(dplyr)
library(jtools)

# Generate a new emmeans object for the second interaction
emm2 <- emmeans(model_hit_img_mus_VA_fx, 
                ~ Valence_Sel_fx * val_mus_fx * arous_mus_fx, 
                type = "response", 
                infer = c(TRUE, TRUE))
```

```
## NOTE: Results may be misleading due to involvement in interactions
```

```
# Now rename the effect-coded levels
emm2@grid$Valence_Sel_fx <- factor(ifelse(emm2@grid$Valence_Sel_fx == 0.5, "Pos", "Neg"))
emm2@grid$val_mus_fx     <- factor(ifelse(emm2@grid$val_mus_fx == 0.5, "Pos", "Neg"))
emm2@grid$arous_mus_fx   <- factor(ifelse(emm2@grid$arous_mus_fx == 0.5, "High", "Low"))

# Plot as before
plot_emm2 <- plot(emm2, type = "response", comparisons = TRUE)

# Adjust colors/lines just like in the first plot
plot_emm2$layers[[3]]$aes_params$colour <- "grey30"
plot_emm2$layers[[3]]$aes_params$linetype <- "dotted"
plot_emm2$layers[[4]]$aes_params$colour <- "grey30"
plot_emm2$layers[[4]]$aes_params$linetype <- "dotted"
plot_emm2$layers[[1]]$aes_params$colour <- "black"
plot_emm2$layers[[2]]$aes_params$colour <- "black"

# Apply theme and labels
plot_emm2_modified <- plot_emm2 +
  theme_apa() +
  theme(
    panel.border = element_blank(),
    axis.line = element_line(color = "black"),
    axis.line.y.right = element_blank(),
    axis.line.x.top = element_blank()
  ) +
  labs(
    x = "Probability of correct recognition (Hit)",
    y = "Valence Img. x Valence Mus. x Arousal Mus."
  )

plot_hit_val_mus_arous_fx <- plot_emm2_modified

plot_hit_val_mus_arous_fx
```

### Emmeans Valence\_Sel × val\_mus

```
# 3. Valence_Sel × val_mus
emm3 <- emmeans(model_hit_img_mus_VA_fx, 
                ~ Valence_Sel_fx * val_mus_fx, 
                type = "response", 
                infer = c(TRUE, TRUE))
```

```
## NOTE: Results may be misleading due to involvement in interactions
```

```
plot(emm3,comparisons = TRUE)
```

```
contr3 <- contrast(emm3, method = "pairwise", adjust = "bonferroni") %>%
  as.data.frame() %>%
  filter(p.value < 0.05)

contr3
```

```
##  contrast                                                                
##  (Valence_Sel_fx-0.5 val_mus_fx-0.5) / (Valence_Sel_fx0.5 val_mus_fx-0.5)
##  odds.ratio     SE  df null z.ratio p.value
##        1.24 0.0724 Inf    1   3.741  0.0011
## 
## Results are averaged over the levels of: Arousal_Sel_fx, arous_mus_fx 
## P value adjustment: bonferroni method for 6 tests 
## Tests are performed on the log odds ratio scale
```

## DPrime Model

#### Create music valence and music arousal variable

```
data_agg2$val_mus <- ifelse(data_agg2$MusicAffect %in% c("happy", "tender"), 1, 0)  # positive valence
data_agg2$arous_mus <- ifelse(data_agg2$MusicAffect %in% c("happy", "fear"), 1, 0)  # high arousal

# Check mapping
#unique(data_agg2[, c("MusicAffect", "val_mus", "arous_mus")])


data_agg2$val_mus <- relevel(as.factor(data_agg2$val_mus), ref = "1")

data_agg2$arous_mus <- relevel(as.factor(data_agg2$arous_mus), ref = "1")

data_agg2$img_val <- relevel(as.factor(data_agg2$img_val), ref = "1")

data_agg2$img_arousal <- relevel(as.factor(data_agg2$img_arousal), ref = "1")
```

#### Log-shift\_dprime

```
# Shift the data to make all values positive
#http://aloy.github.io/lmeresampler/articles/lmeresampler-vignette.html

min_value <- min(data_agg2$dprime_value_congruence_condition)
data_agg2$shifted_dprime <- data_agg2$dprime_value_congruence_condition - min_value + 1

#log transformation
data_agg2$log_shifted_dprime <- log(data_agg2$shifted_dprime)
```

#### Create Effect Coded Variables

```
# Create effect-coded variables for dprime model
data_agg2$img_val_fx   <- car::recode(data_agg2$img_val, "0 = -0.5; 1 = 0.5")
data_agg2$img_arousal_fx <- car::recode(data_agg2$img_arousal, "0= -0.5; 1=0.5")
data_agg2$val_mus_fx   <- car::recode(data_agg2$val_mus, "0= -0.5; 1=0.5")
data_agg2$arous_mus_fx <- car::recode(data_agg2$arous_mus, "0= -0.5; 1=0.5")
```

### Model Effect Coded

```
# Re-fit the model using effect-coded variables
model_dprime_cong_rev_fx <- lmer(
                                  log_shifted_dprime ~ 
                                  img_val_fx * 
                                  img_arousal_fx *
                                  val_mus_fx * 
                                  arous_mus_fx +
                                  mean_pupil_per_trial +
                                  (1 | Subject),
                                  data = data_agg2)
```

### Bootstrapped Confidence Intervals

```
# Bootstrapped fixed-effect estimates
boot_dprime_rev_fx <- bootstrap(
  model_dprime_cong_rev_fx,
  .f = fixef,
  type = "parametric",
  B = 2000  # increase to 1000+ for stable CIs
)

# Examine results
#summary(boot_dprime_rev_fx)
# confint(boot_dprime_rev)
# We report the normal "norm" bootstrap CIs

print(confint(boot_dprime_rev_fx), n = 17)
```

```
## # A tibble: 51 × 6
##    term                                     estimate   lower   upper type  level
##    <chr>                                       <dbl>   <dbl>   <dbl> <chr> <dbl>
##  1 (Intercept)                               2.25     2.21   2.30    norm   0.95
##  2 img_val_fx0.5                            -0.0501  -0.103  0.00521 norm   0.95
##  3 img_arousal_fx0.5                         0.0445  -0.0118 0.100   norm   0.95
##  4 val_mus_fx0.5                            -0.0503  -0.105  0.00401 norm   0.95
##  5 arous_mus_fx0.5                          -0.0497  -0.105  0.00543 norm   0.95
##  6 mean_pupil_per_trial                      0.0320  -0.0229 0.0871  norm   0.95
##  7 img_val_fx0.5:img_arousal_fx0.5           0.0176  -0.0607 0.0952  norm   0.95
##  8 img_val_fx0.5:val_mus_fx0.5               0.0566  -0.0221 0.132   norm   0.95
##  9 img_arousal_fx0.5:val_mus_fx0.5           0.0543  -0.0223 0.132   norm   0.95
## 10 img_val_fx0.5:arous_mus_fx0.5             0.0357  -0.0430 0.113   norm   0.95
## 11 img_arousal_fx0.5:arous_mus_fx0.5         0.00927 -0.0690 0.0878  norm   0.95
## 12 val_mus_fx0.5:arous_mus_fx0.5             0.108    0.0297 0.185   norm   0.95
## 13 img_val_fx0.5:img_arousal_fx0.5:val_mus… -0.00437 -0.113  0.106   norm   0.95
## 14 img_val_fx0.5:img_arousal_fx0.5:arous_m…  0.00730 -0.102  0.119   norm   0.95
## 15 img_val_fx0.5:val_mus_fx0.5:arous_mus_f… -0.0754  -0.182  0.0355  norm   0.95
## 16 img_arousal_fx0.5:val_mus_fx0.5:arous_m… -0.0920  -0.202  0.0171  norm   0.95
## 17 img_val_fx0.5:img_arousal_fx0.5:val_mus…  0.0675  -0.0864 0.219   norm   0.95
## # ℹ 34 more rows
```

#### Diagnostics

##### Simulate DHARMa Residuals

```
library(DHARMa)
sim_res_dprime_cong_rev <- simulateResiduals(fittedModel = model_dprime_cong_rev_fx, n = 1000)
plot(sim_res_dprime_cong_rev)
```

##### Uniformity Test

```
testUniformity_dprime_cong_rev <- testUniformity(sim_res_dprime_cong_rev)
```

```
testUniformity_dprime_cong_rev
```

```
## 
##  Asymptotic one-sample Kolmogorov-Smirnov test
## 
## data:  simulationOutput$scaledResiduals
## D = 0.14367, p-value < 2.2e-16
## alternative hypothesis: two-sided
```

##### Dispersion Test

```
testDispersion_dprime_cong_rev <- testDispersion(sim_res_dprime_cong_rev)
```

```
testDispersion_dprime_cong_rev
```

```
## 
##  DHARMa nonparametric dispersion test via sd of residuals fitted vs.
##  simulated
## 
## data:  simulationOutput
## dispersion = 0.99604, p-value = 0.846
## alternative hypothesis: two.sided
```

##### Normality Test & Q-Q on raw residuals

```
resid_raw_dprime_cong_rev <- residuals(model_dprime_cong_rev_fx)    # conditional residuals
fitted_raw_dprime_cong_rev <- fitted(model_dprime_cong_rev_fx)
plot(fitted_raw_dprime_cong_rev, resid_raw_dprime_cong_rev, xlab="Fitted", ylab="Residuals"); abline(h=0,col="red")
```

```
shapiro.test(resid_raw_dprime_cong_rev)     # note: large N -> sensitive
```

```
## 
##  Shapiro-Wilk normality test
## 
## data:  resid_raw_dprime_cong_rev
## W = 0.85551, p-value < 2.2e-16
```

```
qqnorm(resid_raw_dprime_cong_rev); qqline(resid_raw_dprime_cong_rev)
```

## Eyteracking Model

### Create Subset

```
complete_data_target_fixated <- complete_data_target %>%
  filter(
    !is.na(fixdur_sum_per_image), fixdur_sum_per_image > 0,
    !is.na(fixdur_avg_per_image), fixdur_avg_per_image > 0,
    MusicAffect != "noaudio"
  ) %>%
  droplevels()
```

### Create valence music and arousal music for subset

```
complete_data_target_fixated$val_mus <- ifelse(complete_data_target_fixated$MusicAffect %in% c("happy", "tender"), 1, 0)  # positive valence
complete_data_target_fixated$arous_mus <- ifelse(complete_data_target_fixated$MusicAffect %in% c("happy", "fear"), 1, 0)  # high arousal

# Check mapping
unique(complete_data_target_fixated[, c("MusicAffect", "val_mus", "arous_mus")])
```

```
##    MusicAffect val_mus arous_mus
## 1         fear       0         1
## 5       tender       1         0
## 10       happy       1         1
## 15         sad       0         0
```

#### LMER Model Total Fixation Duration

##### Set Var as factor

```
complete_data_target_fixated$Valence_Pres <- relevel(as.factor(complete_data_target_fixated$Valence_Pres), ref = "1")

complete_data_target_fixated$Arousal_Pres <- relevel(as.factor(complete_data_target_fixated$Arousal_Pres), ref = "1")

complete_data_target_fixated$val_mus <- relevel(as.factor(complete_data_target_fixated$val_mus), ref = "1")

complete_data_target_fixated$arous_mus <- relevel(as.factor(complete_data_target_fixated$arous_mus), ref = "1")
```

#### Model Fixdur Sum

##### Effect Coding Variables

```
# Effect-code categorical predictors
complete_data_target_fixated$Valence_Pres_fx <- ifelse(complete_data_target_fixated$Valence_Pres == "1",  1, -1)
complete_data_target_fixated$Arousal_Pres_fx <- ifelse(complete_data_target_fixated$Arousal_Pres == "1",  1, -1)
complete_data_target_fixated$val_mus_fx      <- ifelse(complete_data_target_fixated$val_mus == "1",  1, -1)
complete_data_target_fixated$arous_mus_fx    <- ifelse(complete_data_target_fixated$arous_mus == "1",  1, -1)
```

##### Model Effect Coded

```
# Fit the model using effect-coded variables
model_fixdur_sum_log_fx <- lmer(
                                log(fixdur_sum_per_image) ~ 
                                Valence_Pres_fx * 
                                Arousal_Pres_fx *
                                val_mus_fx * 
                                arous_mus_fx + 
                                  mean_pupil_per_trial +
                                (1 | Subject),
                                data = complete_data_target_fixated)
```

#### Diagnostics

##### Simulate DHARMa Residuals

```
library(DHARMa)
sim_res_fixdur_sum <- simulateResiduals(fittedModel = model_fixdur_sum_log_fx, n = 1000)
plot(sim_res_fixdur_sum)
```

##### Uniformity Test

```
testUniformity_fixdur_sum <- testUniformity(sim_res_fixdur_sum)
```

```
testUniformity_fixdur_sum
```

```
## 
##  Asymptotic one-sample Kolmogorov-Smirnov test
## 
## data:  simulationOutput$scaledResiduals
## D = 0.061044, p-value < 2.2e-16
## alternative hypothesis: two-sided
```

##### Dispersion Test

```
testDispersion_fixdur_sum <- testDispersion(sim_res_fixdur_sum)
```

```
testDispersion_fixdur_sum
```

```
## 
##  DHARMa nonparametric dispersion test via sd of residuals fitted vs.
##  simulated
## 
## data:  simulationOutput
## dispersion = 0.98768, p-value = 0.68
## alternative hypothesis: two.sided
```

##### Normality Test & Q-Q on raw residuals

```
# resid_raw_fixdur_sum <- residuals(model_fixdur_sum_log_fx)    # conditional residuals
# fitted_raw_fixdur_sum <- fitted(model_fixdur_sum_log_fx)
# plot(fitted_raw_fixdur_sum, resid_raw_fixdur_sum, xlab="Fitted", ylab="Residuals"); abline(h=0,col="red")
# 
# 
# shapiro.test(resid_raw_fixdur_sum)     # note: large N -> sensitive
# qqnorm(resid_raw_fixdur_sum); qqline(resid_raw_fixdur_sum)

#   Shapiro-Wilk normality test
# 
# data:  resid_raw_fixdur_sum
# W = 0.73856, p-value < 2.2e-16
```

#### Parametric Bootstrap

```
# PARAMETRIC BOOTSTRAP
set.seed(1234)  # for reproducibility

boot_fixdur_sum <- bootstrap(
  model_fixdur_sum_log_fx,
  .f = fixef,          # extract fixed effects
  type = "parametric", # parametric bootstrap
  B = 2000             # number of bootstrap samples 
)

# View bootstrapped confidence intervals
ci_fixdur_sum <- confint(boot_fixdur_sum, type = "norm")  # normal-based CIs
print(ci_fixdur_sum, n = 20)
```

```
## # A tibble: 17 × 6
##    term                                   estimate    lower    upper type  level
##    <chr>                                     <dbl>    <dbl>    <dbl> <chr> <dbl>
##  1 (Intercept)                             6.08     6.00e+0  6.16    norm   0.95
##  2 Valence_Pres_fx                        -0.0113  -3.03e-2  0.00771 norm   0.95
##  3 Arousal_Pres_fx                         0.0562   3.74e-2  0.0748  norm   0.95
##  4 val_mus_fx                              0.0155  -3.98e-3  0.0350  norm   0.95
##  5 arous_mus_fx                           -0.0251  -4.47e-2 -0.00584 norm   0.95
##  6 mean_pupil_per_trial                    0.0974  -1.74e-2  0.213   norm   0.95
##  7 Valence_Pres_fx:Arousal_Pres_fx         0.00730 -1.24e-2  0.0268  norm   0.95
##  8 Valence_Pres_fx:val_mus_fx             -0.00595 -2.47e-2  0.0132  norm   0.95
##  9 Arousal_Pres_fx:val_mus_fx              0.00541 -1.37e-2  0.0248  norm   0.95
## 10 Valence_Pres_fx:arous_mus_fx           -0.0158  -3.53e-2  0.00303 norm   0.95
## 11 Arousal_Pres_fx:arous_mus_fx           -0.00923 -2.84e-2  0.00993 norm   0.95
## 12 val_mus_fx:arous_mus_fx                 0.0117  -7.17e-3  0.0313  norm   0.95
## 13 Valence_Pres_fx:Arousal_Pres_fx:val_m…  0.00698 -1.17e-2  0.0263  norm   0.95
## 14 Valence_Pres_fx:Arousal_Pres_fx:arous… -0.00652 -2.55e-2  0.0124  norm   0.95
## 15 Valence_Pres_fx:val_mus_fx:arous_mus_…  0.0176  -8.78e-4  0.0365  norm   0.95
## 16 Arousal_Pres_fx:val_mus_fx:arous_mus_…  0.00343 -1.53e-2  0.0226  norm   0.95
## 17 Valence_Pres_fx:Arousal_Pres_fx:val_m… -0.0119  -3.03e-2  0.00647 norm   0.95
```

```
# Optional: tidy output for tables
ci_fixdur_df_sum <- as.data.frame(ci_fixdur_sum) %>%
  rename(estimate = estimate, lower = lower, upper = upper)
ci_fixdur_df_sum
```

```
##                                                       term     estimate
## 1                                              (Intercept)  6.077999168
## 2                                          Valence_Pres_fx -0.011269611
## 3                                          Arousal_Pres_fx  0.056178705
## 4                                               val_mus_fx  0.015485254
## 5                                             arous_mus_fx -0.025104315
## 6                                     mean_pupil_per_trial  0.097435430
## 7                          Valence_Pres_fx:Arousal_Pres_fx  0.007302092
## 8                               Valence_Pres_fx:val_mus_fx -0.005952400
## 9                               Arousal_Pres_fx:val_mus_fx  0.005412722
## 10                            Valence_Pres_fx:arous_mus_fx -0.015778929
## 11                            Arousal_Pres_fx:arous_mus_fx -0.009229301
## 12                                 val_mus_fx:arous_mus_fx  0.011700411
## 13              Valence_Pres_fx:Arousal_Pres_fx:val_mus_fx  0.006977502
## 14            Valence_Pres_fx:Arousal_Pres_fx:arous_mus_fx -0.006520577
## 15                 Valence_Pres_fx:val_mus_fx:arous_mus_fx  0.017617398
## 16                 Arousal_Pres_fx:val_mus_fx:arous_mus_fx  0.003428874
## 17 Valence_Pres_fx:Arousal_Pres_fx:val_mus_fx:arous_mus_fx -0.011945681
##            lower        upper type level
## 1   5.9993588774  6.156052273 norm  0.95
## 2  -0.0302550107  0.007707707 norm  0.95
## 3   0.0373917147  0.074785273 norm  0.95
## 4  -0.0039849806  0.035043468 norm  0.95
## 5  -0.0447006082 -0.005838829 norm  0.95
## 6  -0.0173690197  0.213395121 norm  0.95
## 7  -0.0124070387  0.026782403 norm  0.95
## 8  -0.0246675148  0.013184631 norm  0.95
## 9  -0.0137429387  0.024818770 norm  0.95
## 10 -0.0353417988  0.003033023 norm  0.95
## 11 -0.0284174970  0.009933848 norm  0.95
## 12 -0.0071708306  0.031338341 norm  0.95
## 13 -0.0117379775  0.026270475 norm  0.95
## 14 -0.0254957288  0.012358795 norm  0.95
## 15 -0.0008776567  0.036526349 norm  0.95
## 16 -0.0153353464  0.022553678 norm  0.95
## 17 -0.0302841070  0.006465862 norm  0.95
```

#### Summary

```
# Summary
summary(model_fixdur_sum_log_fx)
```

```
## Linear mixed model fit by REML. t-tests use Satterthwaite's method [
## lmerModLmerTest]
## Formula: log(fixdur_sum_per_image) ~ Valence_Pres_fx * Arousal_Pres_fx *  
##     val_mus_fx * arous_mus_fx + mean_pupil_per_trial + (1 | Subject)
##    Data: complete_data_target_fixated
## 
## REML criterion at convergence: 13035.5
## 
## Scaled residuals: 
##     Min      1Q  Median      3Q     Max 
## -4.2276 -0.5522  0.1078  0.6781  4.0245 
## 
## Random effects:
##  Groups   Name        Variance Std.Dev.
##  Subject  (Intercept) 0.05936  0.2436  
##  Residual             0.54253  0.7366  
## Number of obs: 5759, groups:  Subject, 41
## 
## Fixed effects:
##                                                           Estimate Std. Error
## (Intercept)                                              6.078e+00  3.982e-02
## Valence_Pres_fx                                         -1.127e-02  9.732e-03
## Arousal_Pres_fx                                          5.618e-02  9.737e-03
## val_mus_fx                                               1.549e-02  9.862e-03
## arous_mus_fx                                            -2.510e-02  9.928e-03
## mean_pupil_per_trial                                     9.743e-02  5.940e-02
## Valence_Pres_fx:Arousal_Pres_fx                          7.302e-03  9.732e-03
## Valence_Pres_fx:val_mus_fx                              -5.952e-03  9.729e-03
## Arousal_Pres_fx:val_mus_fx                               5.413e-03  9.733e-03
## Valence_Pres_fx:arous_mus_fx                            -1.578e-02  9.733e-03
## Arousal_Pres_fx:arous_mus_fx                            -9.229e-03  9.737e-03
## val_mus_fx:arous_mus_fx                                  1.170e-02  1.001e-02
## Valence_Pres_fx:Arousal_Pres_fx:val_mus_fx               6.978e-03  9.735e-03
## Valence_Pres_fx:Arousal_Pres_fx:arous_mus_fx            -6.521e-03  9.743e-03
## Valence_Pres_fx:val_mus_fx:arous_mus_fx                  1.762e-02  9.736e-03
## Arousal_Pres_fx:val_mus_fx:arous_mus_fx                  3.429e-03  9.732e-03
## Valence_Pres_fx:Arousal_Pres_fx:val_mus_fx:arous_mus_fx -1.195e-02  9.739e-03
##                                                                 df t value
## (Intercept)                                              3.893e+01 152.634
## Valence_Pres_fx                                          5.705e+03  -1.158
## Arousal_Pres_fx                                          5.706e+03   5.769
## val_mus_fx                                               5.725e+03   1.570
## arous_mus_fx                                             5.727e+03  -2.529
## mean_pupil_per_trial                                     4.922e+03   1.640
## Valence_Pres_fx:Arousal_Pres_fx                          5.706e+03   0.750
## Valence_Pres_fx:val_mus_fx                               5.705e+03  -0.612
## Arousal_Pres_fx:val_mus_fx                               5.706e+03   0.556
## Valence_Pres_fx:arous_mus_fx                             5.706e+03  -1.621
## Arousal_Pres_fx:arous_mus_fx                             5.707e+03  -0.948
## val_mus_fx:arous_mus_fx                                  5.735e+03   1.168
## Valence_Pres_fx:Arousal_Pres_fx:val_mus_fx               5.706e+03   0.717
## Valence_Pres_fx:Arousal_Pres_fx:arous_mus_fx             5.707e+03  -0.669
## Valence_Pres_fx:val_mus_fx:arous_mus_fx                  5.706e+03   1.810
## Arousal_Pres_fx:val_mus_fx:arous_mus_fx                  5.706e+03   0.352
## Valence_Pres_fx:Arousal_Pres_fx:val_mus_fx:arous_mus_fx  5.707e+03  -1.227
##                                                         Pr(>|t|)    
## (Intercept)                                              < 2e-16 ***
## Valence_Pres_fx                                           0.2469    
## Arousal_Pres_fx                                         8.38e-09 ***
## val_mus_fx                                                0.1164    
## arous_mus_fx                                              0.0115 *  
## mean_pupil_per_trial                                      0.1010    
## Valence_Pres_fx:Arousal_Pres_fx                           0.4531    
## Valence_Pres_fx:val_mus_fx                                0.5407    
## Arousal_Pres_fx:val_mus_fx                                0.5781    
## Valence_Pres_fx:arous_mus_fx                              0.1050    
## Arousal_Pres_fx:arous_mus_fx                              0.3433    
## val_mus_fx:arous_mus_fx                                   0.2427    
## Valence_Pres_fx:Arousal_Pres_fx:val_mus_fx                0.4736    
## Valence_Pres_fx:Arousal_Pres_fx:arous_mus_fx              0.5034    
## Valence_Pres_fx:val_mus_fx:arous_mus_fx                   0.0704 .  
## Arousal_Pres_fx:val_mus_fx:arous_mus_fx                   0.7246    
## Valence_Pres_fx:Arousal_Pres_fx:val_mus_fx:arous_mus_fx   0.2201    
## ---
## Signif. codes:  0 '***' 0.001 '**' 0.01 '*' 0.05 '.' 0.1 ' ' 1
```

```
## 
## Correlation matrix not shown by default, as p = 17 > 12.
## Use print(x, correlation=TRUE)  or
##     vcov(x)        if you need it
```

#### ANOVA

```
Anova(model_fixdur_sum_log_fx, type = 2)
```

```
## Analysis of Deviance Table (Type II Wald chisquare tests)
## 
## Response: log(fixdur_sum_per_image)
##                                                           Chisq Df Pr(>Chisq)
## Valence_Pres_fx                                          1.4116  1    0.23479
## Arousal_Pres_fx                                         33.5881  1  6.811e-09
## val_mus_fx                                               2.5576  1    0.10976
## arous_mus_fx                                             6.2016  1    0.01276
## mean_pupil_per_trial                                     2.6902  1    0.10097
## Valence_Pres_fx:Arousal_Pres_fx                          0.5719  1    0.44950
## Valence_Pres_fx:val_mus_fx                               0.3499  1    0.55418
## Arousal_Pres_fx:val_mus_fx                               0.3305  1    0.56539
## Valence_Pres_fx:arous_mus_fx                             2.5048  1    0.11350
## Arousal_Pres_fx:arous_mus_fx                             0.9169  1    0.33828
## val_mus_fx:arous_mus_fx                                  1.4065  1    0.23564
## Valence_Pres_fx:Arousal_Pres_fx:val_mus_fx               0.4920  1    0.48303
## Valence_Pres_fx:Arousal_Pres_fx:arous_mus_fx             0.4955  1    0.48148
## Valence_Pres_fx:val_mus_fx:arous_mus_fx                  3.1968  1    0.07378
## Arousal_Pres_fx:val_mus_fx:arous_mus_fx                  0.1192  1    0.72995
## Valence_Pres_fx:Arousal_Pres_fx:val_mus_fx:arous_mus_fx  1.5044  1    0.22000
##                                                            
## Valence_Pres_fx                                            
## Arousal_Pres_fx                                         ***
## val_mus_fx                                                 
## arous_mus_fx                                            *  
## mean_pupil_per_trial                                       
## Valence_Pres_fx:Arousal_Pres_fx                            
## Valence_Pres_fx:val_mus_fx                                 
## Arousal_Pres_fx:val_mus_fx                                 
## Valence_Pres_fx:arous_mus_fx                               
## Arousal_Pres_fx:arous_mus_fx                               
## val_mus_fx:arous_mus_fx                                    
## Valence_Pres_fx:Arousal_Pres_fx:val_mus_fx                 
## Valence_Pres_fx:Arousal_Pres_fx:arous_mus_fx               
## Valence_Pres_fx:val_mus_fx:arous_mus_fx                 .  
## Arousal_Pres_fx:val_mus_fx:arous_mus_fx                    
## Valence_Pres_fx:Arousal_Pres_fx:val_mus_fx:arous_mus_fx    
## ---
## Signif. codes:  0 '***' 0.001 '**' 0.01 '*' 0.05 '.' 0.1 ' ' 1
```

### LMER Model Average Fixation Duration

#### Set var as factor

```
complete_data_target_fixated$Valence_Pres <- relevel(as.factor(complete_data_target_fixated$Valence_Pres), ref = "1")

complete_data_target_fixated$Arousal_Pres <- relevel(as.factor(complete_data_target_fixated$Arousal_Pres), ref = "1")

complete_data_target_fixated$val_mus <- relevel(as.factor(complete_data_target_fixated$val_mus), ref = "1")

complete_data_target_fixated$arous_mus <- relevel(as.factor(complete_data_target_fixated$arous_mus), ref = "1")
```

#### Model Fixdur Avg

##### Model effect coded

```
# Fit linear mixed model on log-transformed DV
model_fixdur_avg_log <- lmer(
                                log(fixdur_avg_per_image) ~ 
                                Valence_Pres_fx * 
                                Arousal_Pres_fx *
                                val_mus_fx * 
                                arous_mus_fx + 
                                mean_pupil_per_trial +
                                (1 | Subject),
                                data = complete_data_target_fixated)
```

#### Bootstrapped Confidence Intervals

```
boot_fixdur_avg <- bootstrap(
  model_fixdur_avg_log,
  .f = fixef,          # extract fixed effects
  type = "parametric", # parametric bootstrap
  B = 2000             # number of bootstrap samples 
)

# View bootstrapped confidence intervals
ci_fixdur_avg <- confint(boot_fixdur_avg, type = "norm")  # normal-based CIs
print(ci_fixdur_avg, n = 20)
```

```
## # A tibble: 17 × 6
##    term                                   estimate    lower    upper type  level
##    <chr>                                     <dbl>    <dbl>    <dbl> <chr> <dbl>
##  1 (Intercept)                             5.63     5.52     5.74    norm   0.95
##  2 Valence_Pres_fx                        -0.0110  -0.0283   0.00598 norm   0.95
##  3 Arousal_Pres_fx                         0.0439   0.0269   0.0610  norm   0.95
##  4 val_mus_fx                              0.00665 -0.0105   0.0239  norm   0.95
##  5 arous_mus_fx                           -0.0344  -0.0524  -0.0164  norm   0.95
##  6 mean_pupil_per_trial                    0.0745  -0.0287   0.180   norm   0.95
##  7 Valence_Pres_fx:Arousal_Pres_fx         0.00224 -0.0152   0.0197  norm   0.95
##  8 Valence_Pres_fx:val_mus_fx              0.00358 -0.0133   0.0210  norm   0.95
##  9 Arousal_Pres_fx:val_mus_fx              0.00525 -0.0117   0.0223  norm   0.95
## 10 Valence_Pres_fx:arous_mus_fx           -0.0140  -0.0316   0.00348 norm   0.95
## 11 Arousal_Pres_fx:arous_mus_fx           -0.00806 -0.0253   0.00911 norm   0.95
## 12 val_mus_fx:arous_mus_fx                 0.00927 -0.00860  0.0270  norm   0.95
## 13 Valence_Pres_fx:Arousal_Pres_fx:val_m…  0.00572 -0.0116   0.0227  norm   0.95
## 14 Valence_Pres_fx:Arousal_Pres_fx:arous… -0.00346 -0.0214   0.0142  norm   0.95
## 15 Valence_Pres_fx:val_mus_fx:arous_mus_…  0.0154  -0.00214  0.0325  norm   0.95
## 16 Arousal_Pres_fx:val_mus_fx:arous_mus_…  0.00239 -0.0149   0.0196  norm   0.95
## 17 Valence_Pres_fx:Arousal_Pres_fx:val_m… -0.00938 -0.0268   0.00854 norm   0.95
```

#### Diagnostics

##### Simulate DHARMa Residuals

```
sim_res_fixdur_avg <- simulateResiduals(fittedModel = model_fixdur_avg_log, n = 1000)
plot(sim_res_fixdur_avg)
```

##### Uniformity Test

```
testUniformity_fixdur_avg <- testUniformity(sim_res_fixdur_avg)
```

```
testUniformity_fixdur_avg
```

```
## 
##  Asymptotic one-sample Kolmogorov-Smirnov test
## 
## data:  simulationOutput$scaledResiduals
## D = 0.059429, p-value < 2.2e-16
## alternative hypothesis: two-sided
```

##### Dispersion Test

```
testDispersion_fixdur <- testDispersion(sim_res_fixdur_avg)
```

```
testDispersion_fixdur
```

```
## 
##  DHARMa nonparametric dispersion test via sd of residuals fitted vs.
##  simulated
## 
## data:  simulationOutput
## dispersion = 0.97449, p-value = 0.668
## alternative hypothesis: two.sided
```

##### Normality Test & Q-Q on raw residuals

```
# resid_raw_fixdur_avg <- residuals(model_fixdur_avg_log)    # conditional residuals
# fitted_raw_fixdur_avg <- fitted(model_fixdur_avg_log)
# plot(fitted_raw_fixdur_avg, resid_raw_fixdur_avg, xlab="Fitted", ylab="Residuals"); abline(h=0,col="red")
# 
# 
# shapiro.test(resid_raw_fixdur_avg)     
# qqnorm(resid_raw_fixdur_avg); qqline(resid_raw_fixdur_avg)

# Shapiro-Wilk normality test
# 
# data:  resid_raw_fixdur_avg
# W = 0.99254, p-value = 1.033e-09
```

#### Summary

```
# # Summary is on log scale
# summary(model_fixdur_avg_log)
```

#### ANOVA

```
# Anova(model_fixdur_avg_log, type = 2) # Dont use diagnostics are off
```

### ICC

```
icc(model_fixdur_avg_log)
```

```
## # Intraclass Correlation Coefficient
## 
##     Adjusted ICC: 0.217
##   Unadjusted ICC: 0.216
```

### R2

```
r2(model_fixdur_avg_log)
```

```
## # R2 for Mixed Models
## 
##   Conditional R2: 0.223
##      Marginal R2: 0.007
```

#### Emmean Arousal Pres

```
emm_model_fixdur_avg_log <- emmeans(model_fixdur_avg_log, ~ Arousal_Pres_fx, type = "response")
```

```
## Note: D.f. calculations have been disabled because the number of observations exceeds 3000.
## To enable adjustments, add the argument 'pbkrtest.limit = 5759' (or larger)
## [or, globally, 'set emm_options(pbkrtest.limit = 5759)' or larger];
## but be warned that this may result in large computation time and memory use.
```

```
## Note: D.f. calculations have been disabled because the number of observations exceeds 3000.
## To enable adjustments, add the argument 'lmerTest.limit = 5759' (or larger)
## [or, globally, 'set emm_options(lmerTest.limit = 5759)' or larger];
## but be warned that this may result in large computation time and memory use.
```

```
## NOTE: Results may be misleading due to involvement in interactions
```

```
summary(emm_model_fixdur_avg_log)
```

```
##  Arousal_Pres_fx response   SE  df asymp.LCL asymp.UCL
##               -1      266 15.1 Inf       238       297
##                1      290 16.5 Inf       260       324
## 
## Results are averaged over the levels of: Valence_Pres_fx, val_mus_fx, arous_mus_fx 
## Degrees-of-freedom method: asymptotic 
## Confidence level used: 0.95 
## Intervals are back-transformed from the log scale
```

```
# Plot estimated marginal means with comparisons
 plot(emm_model_fixdur_avg_log, comparisons = TRUE)
```

```
# Perform pairwise comparisons
pairwise_comparisons_emm_model_fixdur_avg_log <- pairs(emm_model_fixdur_avg_log)  

# Extract pairwise comparisons 
pairwise_summary_model_fixdur_avg_log <- summary(pairwise_comparisons_emm_model_fixdur_avg_log, infer = TRUE, adjust = "sidak")

# Significant Pairwise Comparisons
significant_comparisons_emm_model_fixdur_avg_log <- pairwise_summary_model_fixdur_avg_log %>%
  filter(p.value < 0.05)  # Filter for significant comparisons

# Print or notify
if (nrow(significant_comparisons_emm_model_fixdur_avg_log) > 0) {
  print(significant_comparisons_emm_model_fixdur_avg_log)
} else {
  cat("No significant comparisons found.\n")
}
```

```
##  contrast                               ratio     SE  df asymp.LCL asymp.UCL
##  (Arousal_Pres_fx-1) / Arousal_Pres_fx1 0.916 0.0161 Inf     0.885     0.948
##  null z.ratio p.value
##     1  -4.993  <.0001
## 
## Results are averaged over the levels of: Valence_Pres_fx, val_mus_fx, arous_mus_fx 
## Degrees-of-freedom method: asymptotic 
## Confidence level used: 0.95 
## Intervals are back-transformed from the log scale 
## Tests are performed on the log scale
```

#### Emmeans Valence Music

```
emm_model_fixdur_avg_log_val_mus <- emmeans(model_fixdur_avg_log, ~ val_mus_fx, type = "response")
```

```
## Note: D.f. calculations have been disabled because the number of observations exceeds 3000.
## To enable adjustments, add the argument 'pbkrtest.limit = 5759' (or larger)
## [or, globally, 'set emm_options(pbkrtest.limit = 5759)' or larger];
## but be warned that this may result in large computation time and memory use.
```

```
## Note: D.f. calculations have been disabled because the number of observations exceeds 3000.
## To enable adjustments, add the argument 'lmerTest.limit = 5759' (or larger)
## [or, globally, 'set emm_options(lmerTest.limit = 5759)' or larger];
## but be warned that this may result in large computation time and memory use.
```

```
## NOTE: Results may be misleading due to involvement in interactions
```

```
summary(emm_model_fixdur_avg_log_val_mus)
```

```
##  val_mus_fx response   SE  df asymp.LCL asymp.UCL
##          -1      276 15.7 Inf       247       308
##           1      279 15.9 Inf       250       312
## 
## Results are averaged over the levels of: Valence_Pres_fx, Arousal_Pres_fx, arous_mus_fx 
## Degrees-of-freedom method: asymptotic 
## Confidence level used: 0.95 
## Intervals are back-transformed from the log scale
```

```
# Plot estimated marginal means with comparisons
 plot(emm_model_fixdur_avg_log_val_mus, comparisons = TRUE)
```

```
# Perform pairwise comparisons
pairwise_comparisons_emm_model_fixdur_avg_log_val_mus <- pairs(emm_model_fixdur_avg_log_val_mus)  

# Extract pairwise comparisons 
pairwise_summary_model_fixdur_avg_log_val_mus <- summary(pairwise_comparisons_emm_model_fixdur_avg_log_val_mus, infer = TRUE, adjust = "sidak")

# Significant Pairwise Comparisons
significant_comparisons_emm_model_fixdur_avg_log_val_mus <- pairwise_summary_model_fixdur_avg_log_val_mus %>%
  filter(p.value < 0.05)  # Filter for significant comparisons

# Print or notify
if (nrow(significant_comparisons_emm_model_fixdur_avg_log_val_mus) > 0) {
  print(significant_comparisons_emm_model_fixdur_avg_log_val_mus)
} else {
  cat("No significant comparisons found.\n") }
```

```
## No significant comparisons found.
```

### Number of Fixations Model

```
model_nfix <- glmer(Number_of_fix_to_image_per_trial ~ 
                      Valence_Pres_fx * 
                      Arousal_Pres_fx * 
                      val_mus_fx * 
                      arous_mus_fx +  
                      (1|Subject),
                    family = poisson(link="log"),
                    data = complete_data_target_fixated)
```

#### Diagnostics

```
sim_res_nfix <- simulateResiduals(fittedModel = model_nfix, n = 1000)
plot(sim_res_nfix)
```

#### Uniformity Test

```
testUniformity(sim_res_nfix)
```

```
## 
##  Asymptotic one-sample Kolmogorov-Smirnov test
## 
## data:  simulationOutput$scaledResiduals
## D = 0.16317, p-value < 2.2e-16
## alternative hypothesis: two-sided
```

#### Dispersion Test

```
testDispersion(sim_res_nfix)
```

```
## 
##  DHARMa nonparametric dispersion test via sd of residuals fitted vs.
##  simulated
## 
## data:  simulationOutput
## dispersion = 0.53848, p-value < 2.2e-16
## alternative hypothesis: two.sided
```

#### Zero-Inflation Test

```
testZeroInflation(sim_res_nfix)
```

```
## 
##  DHARMa zero-inflation test via comparison to expected zeros with
##  simulation under H0 = fitted model
## 
## data:  simulationOutput
## ratioObsSim = 0, p-value < 2.2e-16
## alternative hypothesis: two.sided
```

#### Outlier Test

```
testOutliers(sim_res_nfix,type = 'bootstrap')
```

```
## 
##  DHARMa bootstrapped outlier test
## 
## data:  sim_res_nfix
## outliers at both margin(s) = 3, observations = 5759, p-value = 0.64
## alternative hypothesis: two.sided
##  percent confidence interval:
##  0.0001736413 0.0017364126
## sample estimates:
## outlier frequency (expected: 0.000859524222955374 ) 
##                                        0.0005209238
```

#### Residuals vs. Fitted

```
plot(fitted(model_nfix), residuals(model_nfix, type = "pearson"))
abline(h = 0, col = "red")
```

### Negative Binomial

```
# library(glmmTMB)
# 
# model_nfix_nb <- glmmTMB(
#   Number_of_fix_to_image_per_trial ~ 
#     Valence_Pres_fx * 
#     Arousal_Pres_fx *
#     val_mus_fx *
#     arous_mus_fx +
#     (1|Subject),
#   family = nbinom2, 
#   data = complete_data_target_fixated)
# 
# sim_res_nb <- simulateResiduals(model_nfix_nb, n = 1000)
# plot(sim_res_nb)
# testDispersion(sim_res_nb)
# testZeroInflation(sim_res_nb)
```

We explored modelling the number of fixations per image using Poisson
mixed models, but model diagnostics (DHARMa tests for dispersion and
zero structure) indicated severe underdispersion that could not be
resolved with alternative model specifications. Therefore, fixation
count analyses were not included in the final report.

## Simulation Based Power Analysis

### Hit Model Power Analysis

#### PowerCurve Hit Model four-way interaction

Calculate power at our n = 41 and at a higher n = 100 for an
estimation of required n for 80% power

```
# # Our original fitted model
# model_hit <- model_hit_img_mus_VA_fx
# 
# # Name of the 4-way interaction
# target <- "Valence_Sel_fx:Arousal_Sel_fx:val_mus_fx:arous_mus_fx"
# 
# # Set medium effect size
# # fixef(model_hit)[target] <- 0.5
# model_sim_hit_fourway <- extend(model_hit, along = "Subject", n = 100)
# fixef(model_sim_hit_fourway)[target] <- 0.5
# 
# # Run power curve by increasing number of subjects
# power_curve_fourway_hit_v2 <- powerCurve(model_sim_hit_fourway,
#                           fixed(target),
#                           along = "Subject",
#                           nsim = 100,   
#                           breaks = c( 100))  # adjusted based on our range
#                           # original breaks 41 (our n),100
# 
# power_curve_fourway_hit_v2

# Save result
# saveRDS(power_curve_fourway_hit_v2, "power_curve_fourway_hit_v2.rds")

# # Load result
#  pc_hit_fourway_v2 <- readRDS("power_curve_fourway_hit_v2.rds")

# print(pc_hit_fourway_v2)

## Power Curve nsim = 100 for n = 100
# Calculating power at 1 sample sizes along Subject
# Power for predictor 'Valence_Sel_fx:Arousal_Sel_fx:val_mus_fx:arous_mus_fx', (95% confidence interval),==============================|
# by largest value of Subject:
#     100: 64.00% (53.79, 73.36) - 28707 rows
#
# Time elapsed: 1 h 14 m 29 s

#########
# This first power calculation accidentally did not extend along subject, i.e. did not increase sample size, but instead computed 1000 simulations for our actual sample size

# Save result
#saveRDS(power_curve_fourway_hit, "power_curve_fourway_hit.rds")

# pc_hit_fourway
# Power for predictor 'Valence_Sel_fx:Arousal_Sel_fx:val_mus_fx:arous_mus_fx', (95% # confidence interval),
# by largest value of Subject:
  #   42: 34.70% (31.75, 37.74) - 11585 rows
   #  NA: 34.70% (31.75, 37.74) - 11585 rows
    # NA: 34.70% (31.75, 37.74) - 11585 rows

# Time elapsed: 15 h 16 m 0 s
 
#### n = 41 ###
## Load first power curve result of n = 41
#pc_hit_fourway_v2 <- readRDS("power_curve_fourway_hit.rds")
# print(pc_hit_fourway_v2)
```

#### Three-way Interaction V Img x A Img x A Mus

```
# # Here we compute the power for the significant three-way interaction from the hit model which had the larger ChiSquare of the two.
# 
# # Our original fitted model
# model_hit <- model_hit_img_mus_VA_fx
# 
# # Name of the 3-way interaction [target]
# target_three_way_ViAiAm <- "Valence_Sel_fx:Arousal_Sel_fx:arous_mus_fx"
# 
# # Set medium effect size
# model_sim_hit_threeway_ViAiAm <- extend(model_hit, along = "Subject", n = 100)
# fixef(model_sim_hit_threeway_ViAiAm)[target_three_way_ViAiAm] <- 0.5
# 
# # Run power curve by increasing number of subjects
# power_curve_threeway_hit_ViAiAm <- powerCurve(model_sim_hit_threeway_ViAiAm,
#                           fixed(target_three_way_ViAiAm),
#                           along = "Subject",
#                           nsim = 100,  
#                           breaks = c(41, 100))  # adjusted based on our range
#                           # original breaks 41 (our n), 100
# 
# power_curve_threeway_hit_ViAiAm

# Save result
#saveRDS(power_curve_threeway_hit_ViAiAm, "power_curve_threeway_hit_ViAiAm.rds")

# # Load result
#  power_curve_threeway_hit_ViAiAm <- readRDS("power_curve_threeway_hit_ViAiAm.rds")

# print(power_curve_threeway_hit_ViAiAm)


# Power for predictor 'Valence_Sel_fx:Arousal_Sel_fx:arous_mus_fx', (95% confidence interval),
# by largest value of Subject:
#      41: 84.00% (75.32, 90.57) - 11585 rows
#     100: 99.00% (94.55, 99.97) - 28707 rows
# 
# Time elapsed: 1 h 45 m 13 s
```

#### Three-way Interaction V Img V Mus A Mus

```
# Here we compute the power for the significant three-way interaction from the hit model which had the smaller ChiSquare of the two.

# # Our original fitted model
# model_hit <- model_hit_img_mus_VA_fx
# 
# # Name of the 4-way interaction [target]
# target_three_way_ViVmAm <- "Valence_Sel_fx:val_mus_fx:arous_mus_fx"
# 
# # Set medium effect size
# model_sim_hit_threeway_ViVmAm <- extend(model_hit, along = "Subject", n = 100)
# fixef(model_sim_hit_threeway_ViVmAm)[target_three_way_ViVmAm] <- 0.5
# 
# # Run power curve by increasing number of subjects
# power_curve_threeway_hit_ViVmAm <- powerCurve(model_sim_hit_threeway_ViVmAm,
#                           fixed(target_three_way_ViVmAm),
#                           along = "Subject",
#                           nsim = 100,  
#                           breaks = c(41, 100))  # adjusted based on our range
#                           # original breaks 41 (our n), 100
# 
# power_curve_threeway_hit_ViVmAm

# Save result
 #saveRDS(power_curve_threeway_hit_ViVmAm, "power_curve_threeway_hit_ViVmAm.rds")

# # Load result
#  power_curve_threeway_hit_ViVmAm <- readRDS("power_curve_threeway_hit_ViVmAm.rds")

# print(power_curve_threeway_hit_ViVmAm)


# Power for predictor 'Valence_Sel_fx:val_mus_fx:arous_mus_fx', (95% confidence interval),
# by largest value of Subject:
  #   41: 87.00% (78.80, 92.89) - 11585 rows
   # 100: 99.00% (94.55, 99.97) - 28707 rows

# Time elapsed: 1 h 49 m 27 s
```

#### Arousal Image Power

```
# # Name of the fixed effect
# target_arousal_sel <- "Arousal_Sel_fx"
# 
# # Set medium effect size
# fixef(model_hit)[target_arousal_sel] <- 0.5
# 
# # Run power curve by increasing number of subjects
# power_curve_arousal_sel <- powerCurve(model_hit,
#                           fixed(target_arousal_sel),
#                           along = "Subject",
#                           nsim = 100,   
#                           breaks = c(41, 100))  # adjusted based on our range
# 
# power_curve_arousal_sel

# Save result
#saveRDS(power_curve_arousal_sel, "power_curve_arousal_sel.rds")
# 
# # Load result
#  power_curve_arousal_sel <- readRDS("power_curve_arousal_sel.rds")

# print(power_curve_arousal_sel)

# Test run with few simulations
# Calculating power at 3 sample sizes along Subject
# Power for predictor 'Arousal_Sel_fx', (95% confidence interval),=============================================================================================|
# by largest value of Subject:
#      41: 100.0% (69.15, 100.0) - 11167 rows
#      NA: 100.0% (69.15, 100.0) - 11585 rows
#      NA: 100.0% (69.15, 100.0) - 11585 rows

# Time elapsed: 0 h 8 m 12 s
```

#### Valence Image Power

```
# # Name of the fixed effect
# target_valence_sel <- "Valence_Sel_fx"
# 
# # Set medium effect size
# fixef(model_hit)[target_valence_sel] <- 0.5
# 
# # Run power curve by increasing number of subjects
# power_curve_valence_sel <- powerCurve(model_hit,
#                           fixed(target_valence_sel),
#                           along = "Subject",
#                           nsim = 100,  
#                           breaks = c(41, 100))  # adjusted based on our range
# 
# power_curve_valence_sel

# Save result
# saveRDS(power_curve_valence_sel, "power_curve_valence_sel.rds")

# # Load result
# power_curve_valence_sel <- readRDS("power_curve_valence_sel.rds")

# Display reuslt
# print(power_curve_valence_sel)

# Power for predictor 'Valence_Sel_fx', (95% confidence interval),
# by largest value of Subject:
#      42: 100.0% (69.15, 100.0) - 11585 rows
#      NA: 100.0% (69.15, 100.0) - 11585 rows
#      NA: 100.0% (69.15, 100.0) - 11585 rows
# 
# Time elapsed: 0 h 8 m 8 s
```

#### Valence Music Power

```
# # Name of the fixed effect
# target_valence_mus <- "val_mus_fx"
# 
# # Set medium effect size
# fixef(model_hit)[target_valence_mus] <- 0.5
# 
# # Run power curve by increasing number of subjects
# power_curve_valence_mus <- powerCurve(model_hit,
#                           fixed(target_valence_mus),
#                           along = "Subject",
#                           nsim = 1,  
#                           breaks = c(41, 80, 100))  # adjusted based on our range
# 
# power_curve_valence_mus
# 
# # Save result
# # saveRDS(power_curve_valence_mus, "power_curve_valence_mus.rds")
# # Load result
# # pc_val_mus <- readRDS("power_curve_valence_mus.rds")
# 
# pc_val_mus
```

#### Arousal Music Power

```
# # Name of the fixed effect
# target_arous_mus <- "arous_mus_fx"
# 
# # Set medium effect size
# fixef(model_hit)[target_arous_mus] <- 0.5
# 
# # Run power curve by increasing number of subjects
# power_curve_arous_mus <- powerCurve(model_hit,
#                           fixed(target_arous_mus),
#                           along = "Subject",
#                           nsim = 1,   # we need to increase to 1000+ for final
#                           breaks = c(41, 80, 100))  # adjusted based on our range
# 
# power_curve_arous_mus

# Save result
# saveRDS(power_curve_arous_mus, "power_curve_arous_mus.rds")
# Load result
# pc <- readRDS("power_curve_arous_mus.rds")
```

### d’ DPrime Model Power

#### Four-way interaction power

```
# # Name of the 4-way interaction
# target_4_way_dprime <- "img_val_fx0.5:img_arousal_fx0.5:val_mus_fx0.5:arous_mus_fx0.5"
# 
# # Extend subjects
# model_sim_dprime_fourway <- extend(
#   model_dprime_cong_rev_fx, 
#   along = "Subject", 
#   n = 100
# )
# 
# # Set medium effect size
# fixef(model_sim_dprime_fourway)[target_4_way_dprime] <- 0.5
# 
# # Run power curve by increasing number of subjects
# power_curve_4_way_dprime <- powerCurve(
#   model_sim_dprime_fourway,
#   fixed(target_4_way_dprime),
#   along = "Subject",
#   nsim = 100,                
#   breaks = c(41, 100)
# )
# 
# power_curve_4_way_dprime
# 
# # Save
# saveRDS(power_curve_4_way_dprime, "power_curve_4_way_dprime.rds")
# 
# # Load result
# # pc_hit_4-way <- readRDS("power_curve_4_way_dprime.rds")
```

#### Valence x Arousal Music DPrime Power

```
# # Name of the 2-way interaction [target]
# target_twoway_dprime <- "val_mus_fx0.5:arous_mus_fx0.5"
# 
# # Set medium effect size
# model_sim_dprime_mus2 <- extend(model_dprime_cong_rev_fx, along = "Subject", n = 100)
# fixef(model_sim_dprime_mus2)[target_twoway_dprime] <- 0.5
# 
# # Run power curve by increasing number of subjects
# power_curve_two_way_dprime <- powerCurve(model_sim_dprime_mus2,
#                           fixed(target_twoway_dprime),
#                           along = "Subject",
#                           nsim = 100,  
#                           breaks = c(41, 100))  # adjusted based on our range
#                           # original breaks 41 (our n), 100
# 
# power_curve_two_way_dprime
# 
# # Save result
#  saveRDS(power_curve_two_way_dprime, "power_curve_two_way_dprime.rds")
# 
# # Load result
#  power_curve_two_way_dprime <- readRDS("power_curve_two_way_dprime.rds")
# 
# print(power_curve_two_way_dprime)
```

#### Image Valence Power

```
# # Name of the 4-way interaction
# target_img_val_dprime <- "img_val_fx0.5"
# 
# # Set medium effect size
# fixef(model_dprime_cong_rev_fx)[target_img_val_dprime] <- 0.5
# 
# # Run power curve by increasing number of subjects
# power_curve_img_val_dprime <- powerCurve(model_dprime_cong_rev_fx,
#                           fixed(target_img_val_dprime),
#                           along = "Subject",
#                           nsim = 3,   
#                           breaks = c(41, 80, 100))  # adjusted based on our range
# 
# power_curve_img_val_dprime
# 
# # Save result
# saveRDS(power_curve_img_val_dprime, "power_curve_img_val_dprime.rds")
# # Load result
# # pc <- readRDS("power_curve_img_val_dprime.rds")
```

#### Image Arousal Power

```
# # Name of the 4-way interaction
# target_img_arous_dprime <- "img_arousal_fx0.5"
# 
# # Set medium effect size
# fixef(model_dprime_cong_rev_fx)[target_img_arous_dprime] <- 0.5
# 
# # Run power curve by increasing number of subjects
# power_curve_img_arous_dprime <- powerCurve(model_dprime_cong_rev_fx,
#                           fixed(target_img_arous_dprime),
#                           along = "Subject",
#                           nsim = 1000, 
#                           breaks = c(41, 80, 100))  # adjusted based on our range
# 
# power_curve_img_arous_dprime
# 
# # Save result
# saveRDS(power_curve_img_arous_dprime, "power_curve_img_arous_dprime.rds")
# # Load result
# # pc <- readRDS("power_curve_img_arous_dprime.rds")
```

### Power for Fixdur avg

#### Image Arousal

```
# # Name of the Fixed Effect
# target_img_arousal_fixdur <- "Arousal_Pres0"
# 
# # Set medium effect size
# model_fixdur_avg_log_sim <- extend(model_fixdur_avg_log, along = "Subject", n = 100)
# fixef(model_fixdur_avg_log_sim)[target_img_arousal_fixdur] <- 0.5
# 
# # Run power curve by increasing number of subjects
# power_curve_fixdur_avg_img_arousal <- powerCurve(model_fixdur_avg_log_sim,
#                          test = fixed(target_img_arousal_fixdur),
#                           along = "Subject",
#                           nsim = 100,  
#                           breaks = c(41, 100))  # adjusted based on our range
#                           # original breaks 41 (our n), 100
# 
# power_curve_fixdur_avg_img_arousal
# 
# # Save result
#  saveRDS(power_curve_fixdur_avg_img_arousal, "power_curve_fixdur_avg_img_arousal.rds")
# 
# # Load result
#  power_curve_fixdur_avg_img_arousal <- readRDS("power_curve_fixdur_avg_img_arousal.rds")
# 
# print(power_curve_fixdur_avg_img_arousal)
```

# Pre-Test Ratings

## Image Valence

```
# Standardizing the dependent variable
pretest_img_rating$valence_img_pretest_standardized <- scale(pretest_img_rating$valence_img_pretest)


img_pretest_valence_standardized <- lm(valence_img_pretest_standardized ~ valence_binary * arousal_binary, data = pretest_img_rating)

summary(img_pretest_valence_standardized)
```

```
## 
## Call:
## lm(formula = valence_img_pretest_standardized ~ valence_binary * 
##     arousal_binary, data = pretest_img_rating)
## 
## Residuals:
##      Min       1Q   Median       3Q      Max 
## -2.14284 -0.49223  0.09984  0.33308  2.57575 
## 
## Coefficients:
##                               Estimate Std. Error t value Pr(>|t|)    
## (Intercept)                    -0.6912     0.1238  -5.583 2.56e-08 ***
## valence_binary                  0.7370     0.1317   5.598 2.36e-08 ***
## arousal_binary                 -0.2855     0.1266  -2.255   0.0242 *  
## valence_binary:arousal_binary   0.6803     0.1355   5.022 5.41e-07 ***
## ---
## Signif. codes:  0 '***' 0.001 '**' 0.01 '*' 0.05 '.' 0.1 ' ' 1
## 
## Residual standard error: 0.783 on 3164 degrees of freedom
## Multiple R-squared:  0.3876, Adjusted R-squared:  0.387 
## F-statistic: 667.4 on 3 and 3164 DF,  p-value: < 2.2e-16
```

```
# Post-Hoc 
emms_img_pretest_valence_standardized <- emmeans(img_pretest_valence_standardized, pairwise ~ valence_binary * arousal_binary)

# plot(emms_img_pretest_valence_standardized)

# View results of pairwise comparisons
summary(emms_img_pretest_valence_standardized)
```

```
## $emmeans
##  valence_binary arousal_binary  emmean     SE   df lower.CL upper.CL
##               0              0 -0.6912 0.1238 3164  -0.9339   -0.448
##               1              0  0.0458 0.0448 3164  -0.0421    0.134
##               0              1 -0.9767 0.0266 3164  -1.0288   -0.925
##               1              1  0.4406 0.0177 3164   0.4059    0.475
## 
## Confidence level used: 0.95 
## 
## $contrasts
##  contrast                                                          estimate
##  valence_binary0 arousal_binary0 - valence_binary1 arousal_binary0   -0.737
##  valence_binary0 arousal_binary0 - valence_binary0 arousal_binary1    0.286
##  valence_binary0 arousal_binary0 - valence_binary1 arousal_binary1   -1.132
##  valence_binary1 arousal_binary0 - valence_binary0 arousal_binary1    1.023
##  valence_binary1 arousal_binary0 - valence_binary1 arousal_binary1   -0.395
##  valence_binary0 arousal_binary1 - valence_binary1 arousal_binary1   -1.417
##      SE   df t.ratio p.value
##  0.1317 3164  -5.598  <.0001
##  0.1266 3164   2.255  0.1090
##  0.1251 3164  -9.051  <.0001
##  0.0521 3164  19.621  <.0001
##  0.0482 3164  -8.191  <.0001
##  0.0319 3164 -44.384  <.0001
## 
## P value adjustment: tukey method for comparing a family of 4 estimates
```

```
summary(emms_img_pretest_valence_standardized, infer = c(TRUE, TRUE))
```

```
## $emmeans
##  valence_binary arousal_binary  emmean     SE   df lower.CL upper.CL t.ratio
##               0              0 -0.6912 0.1238 3164  -0.9339   -0.448  -5.583
##               1              0  0.0458 0.0448 3164  -0.0421    0.134   1.022
##               0              1 -0.9767 0.0266 3164  -1.0288   -0.925 -36.753
##               1              1  0.4406 0.0177 3164   0.4059    0.475  24.884
##  p.value
##   <.0001
##   0.3067
##   <.0001
##   <.0001
## 
## Confidence level used: 0.95 
## 
## $contrasts
##  contrast                                                          estimate
##  valence_binary0 arousal_binary0 - valence_binary1 arousal_binary0   -0.737
##  valence_binary0 arousal_binary0 - valence_binary0 arousal_binary1    0.286
##  valence_binary0 arousal_binary0 - valence_binary1 arousal_binary1   -1.132
##  valence_binary1 arousal_binary0 - valence_binary0 arousal_binary1    1.023
##  valence_binary1 arousal_binary0 - valence_binary1 arousal_binary1   -0.395
##  valence_binary0 arousal_binary1 - valence_binary1 arousal_binary1   -1.417
##      SE   df lower.CL upper.CL t.ratio p.value
##  0.1317 3164  -1.0755   -0.399  -5.598  <.0001
##  0.1266 3164  -0.0399    0.611   2.255  0.1090
##  0.1251 3164  -1.4533   -0.810  -9.051  <.0001
##  0.0521 3164   0.8886    1.157  19.621  <.0001
##  0.0482 3164  -0.5187   -0.271  -8.191  <.0001
##  0.0319 3164  -1.4995   -1.335 -44.384  <.0001
## 
## Confidence level used: 0.95 
## Conf-level adjustment: tukey method for comparing a family of 4 estimates 
## P value adjustment: tukey method for comparing a family of 4 estimates
```

```
# R² marginal (proportion of variance explained by the fixed effects)
r_squared_img_pretest_valence_standardized <- r.squaredGLMM(img_pretest_valence_standardized)
```

```
## Warning: 'r.squaredGLMM' now calculates a revised statistic. See the help page.
```

```
r_squared_img_pretest_valence_standardized
```

```
##            R2m       R2c
## [1,] 0.3873388 0.3873388
```

## Image Arousal

```
# Standardizing the dependent variable
pretest_img_rating$arousal_img_pretest_standardized <- scale(pretest_img_rating$arousal_img_pretest)


img_pretest_arousal_standardized <- lm(arousal_img_pretest_standardized ~ valence_binary * arousal_binary, data = pretest_img_rating)

summary(img_pretest_arousal_standardized)
```

```
## 
## Call:
## lm(formula = arousal_img_pretest_standardized ~ valence_binary * 
##     arousal_binary, data = pretest_img_rating)
## 
## Residuals:
##     Min      1Q  Median      3Q     Max 
## -1.9917 -0.3604 -0.1522  0.7201  2.2565 
## 
## Coefficients:
##                               Estimate Std. Error t value Pr(>|t|)    
## (Intercept)                   -0.71522    0.15418  -4.639 3.65e-06 ***
## valence_binary                 0.09867    0.16398   0.602    0.547    
## arousal_binary                 0.85780    0.15769   5.440 5.74e-08 ***
## valence_binary:arousal_binary -0.19373    0.16873  -1.148    0.251    
## ---
## Signif. codes:  0 '***' 0.001 '**' 0.01 '*' 0.05 '.' 0.1 ' ' 1
## 
## Residual standard error: 0.9751 on 3164 degrees of freedom
## Multiple R-squared:  0.05004,    Adjusted R-squared:  0.04913 
## F-statistic: 55.55 on 3 and 3164 DF,  p-value: < 2.2e-16
```

```
# Post-Hoc 
emms_img_pretest_arousal_standardized <- emmeans(img_pretest_arousal_standardized, pairwise ~ valence_binary * arousal_binary)

# View results of pairwise comparisons
summary(emms_img_pretest_arousal_standardized)
```

```
## $emmeans
##  valence_binary arousal_binary  emmean     SE   df lower.CL upper.CL
##               0              0 -0.7152 0.1542 3164 -1.01752  -0.4129
##               1              0 -0.6166 0.0558 3164 -0.72603  -0.5071
##               0              1  0.1426 0.0331 3164  0.07769   0.2075
##               1              1  0.0475 0.0221 3164  0.00428   0.0908
## 
## Confidence level used: 0.95 
## 
## $contrasts
##  contrast                                                          estimate
##  valence_binary0 arousal_binary0 - valence_binary1 arousal_binary0  -0.0987
##  valence_binary0 arousal_binary0 - valence_binary0 arousal_binary1  -0.8578
##  valence_binary0 arousal_binary0 - valence_binary1 arousal_binary1  -0.7627
##  valence_binary1 arousal_binary0 - valence_binary0 arousal_binary1  -0.7591
##  valence_binary1 arousal_binary0 - valence_binary1 arousal_binary1  -0.6641
##  valence_binary0 arousal_binary1 - valence_binary1 arousal_binary1   0.0951
##      SE   df t.ratio p.value
##  0.1640 3164  -0.602  0.9315
##  0.1577 3164  -5.440  <.0001
##  0.1557 3164  -4.897  <.0001
##  0.0649 3164 -11.696  <.0001
##  0.0600 3164 -11.062  <.0001
##  0.0398 3164   2.390  0.0791
## 
## P value adjustment: tukey method for comparing a family of 4 estimates
```
